# Total Syntheses of (−)‐Minovincine and (−)‐Aspidofractinine through a Sequence of Cascade Reactions

**DOI:** 10.1002/anie.202004769

**Published:** 2020-06-03

**Authors:** Szilárd Varga, Péter Angyal, Gábor Martin, Orsolya Egyed, Tamás Holczbauer, Tibor Soós

**Affiliations:** ^1^ Institute of Organic Chemistry Research Centre for Natural Sciences 2 Magyar tudósok krt. 1117 Budapest Hungary; ^2^ Instrumentation Center Research Centre for Natural Sciences 2 Magyar tudósok krt. 1117 Budapest Hungary

**Keywords:** alkaloids, asymmetric catalysis, biomimetic synthesis, organocatalysis, total synthesis

## Abstract

We report 8‐step syntheses of (−)‐minovincine and (−)‐aspidofractinine using easily available and inexpensive reagents and catalyst. A key element of the strategy was the utilization of a sequence of cascade reactions to rapidly construct the penta‐ and hexacyclic frameworks. These cascade transformations included organocatalytic Michael‐aldol condensation, a multistep anionic Michael‐S_N_2 cascade reaction, and Mannich reaction interrupted Fischer indolization. To streamline the synthetic routes, we also investigated the deliberate use of steric effect to secure various chemo‐ and regioselective transformations.

## Graphical abstract

**Concise syntheses** of (−)‐minovincine and (−)‐aspidofractinine were achieved using easily available and inexpensive reagents and catalyst. A key element of the strategy was the use of a sequence of cascade reactions to rapidly construct the penta‐ and hexacyclic frameworks. To streamline the synthetic routes, the steric effect was used to secure various chemo‐ and regioselective transformations.

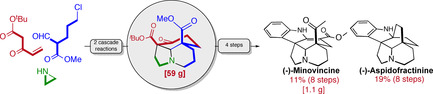

The aspidosperma family of alkaloids continues to attract high interest owing to their medical potential and intriguing chemical structure.^1^ Their stereochemical complexity presents an intrinsic synthetic challenge and their polycyclic, cage‐like structures provide much latitude for advancing different synthetic strategies. As a result, this class of compounds has frequently served as a benchmark target for the development of innovative synthetic methods and strategies.^2^ In particular, aspidospermanes have often provided inspiration for the synthetic community to devise efficient catalytic approaches that allow the stereoselective construction of quaternary carbon stereocenters (C‐5 and C‐12 in Figure 1). The emerging power of these asymmetric methods has not only enriched the aspidosperma chemistry, but also enabled the pursuit of concise synthetic strategies and collective synthesis of structurally related aspidosperma alkaloids.^3^

**Figure 1:**
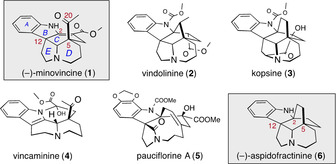
Structure of (−)‐minovincine (**1**), (−)‐aspidofractinine (**6**) and related alkaloids.

There is a distinct aspidosperma alkaloid, (−)‐minovincine (**1**), that possesses a C‐20 oxygenation that is not common within this class of alkaloids. With this subtle but remarkable structural modification, this alkaloid has evolved as a biogenetic springboard toward more complex pleiocarpine‐refractine classes of alkaloids (e.g. **2**, **3** in Figure 1). Additionally, this unique aspidospermane derivative can be transformed to pauciflorine‐4 and eburnane‐type^5^ alkaloids (**4**, **5** in Figure 1) in few chemical steps. Owing to the apparent synthetic significance of minovincine (**1**), it has been an attractive research target since its isolation^6^ in 1962.^7^ Interestingly, and despite the advances of catalytic asymmetric methods, only two enantioselective approaches have been reported by MacMillan^8^ and Nishida.^9^ One rationale for this relative paucity in synthetic routes is the shortcomings in the existing repository of asymmetric methods that can concisely deliver the aspidosperma skeleton with oxidized exocyclic functionality in position C‐5.

Stimulated by the synthetic challenges of (−)‐minovincine (**1**), with its added (bio)synthetic potential, we aimed to develop a concise and scalable route of (−)‐minovincine (**1**). Our hope was also to expand our synthetic strategy toward topologically different minovincine‐derived natural products. Therefore, a refractine‐type alkaloid was also targeted, the hexacyclic (−)‐aspidofractinine (**6**). As an overarching goal, we aimed to devise streamlined synthetic routes that would achieve high levels of “ideality”^10^ and synthetic practicality.^11^ Three interwoven principles directed our synthetic design: 1) to implement a sequence of cascade reactions^12^ for the rapid construction of the penta‐ and hexacyclic frameworks, 2) to minimize protecting‐group manipulations and steer the selectivity of reactions through the steric effect,^13^ and 3) to avoid exotic, toxic, or expensive reagents or catalysts.

Recently, our group developed a strategy for the concise and diastereoselective synthesis of *cis*‐ and *trans*‐decaline subunits of terpenoids.^14^ Key to this strategy was an organocatalyzed Robinson annulation reaction that afforded a chiral enone building block with a quaternary stereogenic center. As an outgrowth of these studies, we were intrigued to develop an analogous chiral building block **7**, which might confer synthetic practicality in aspidospermane chemistry. Our retrosynthetic plan adopted in this investigation is illustrated in Scheme 1. We envisioned tricyclic **8**, a modified Stork's tricyclic ketone,^15^ to serve as an advanced intermediate en route to **1** and **6**. We also expected that the C‐5 ester functionality of **8** can be selectively transformed into the requisite exocyclic keto group to establish the correct functionality in minovincine (**1**). Furthermore, on the grounds of proposed biosynthetic pathway of aspidofractinine (**6**),^16^ our hope was that its cage‐like skeleton might be secured through interception of the C‐2 carbon atom of the transient iminium ion of **9** by its acetyl functionality. Finally, we aspired to construct the desired tricyclic ketone **8** in a concise way to provide opportunities for a scalable synthesis of this advanced intermediate. Therefore, we devised a short synthetic route through the organocatalytic Robinson annulation reaction of **10** and **11** to give multifunctional enone **7** followed by a multistep nucleophilic cascade reaction of aziridine (**12**). We aimed at early introduction of the C‐5 quaternary center and used that center to direct the relative configuration of further functionalization around the C ring.

**Scheme 1:**
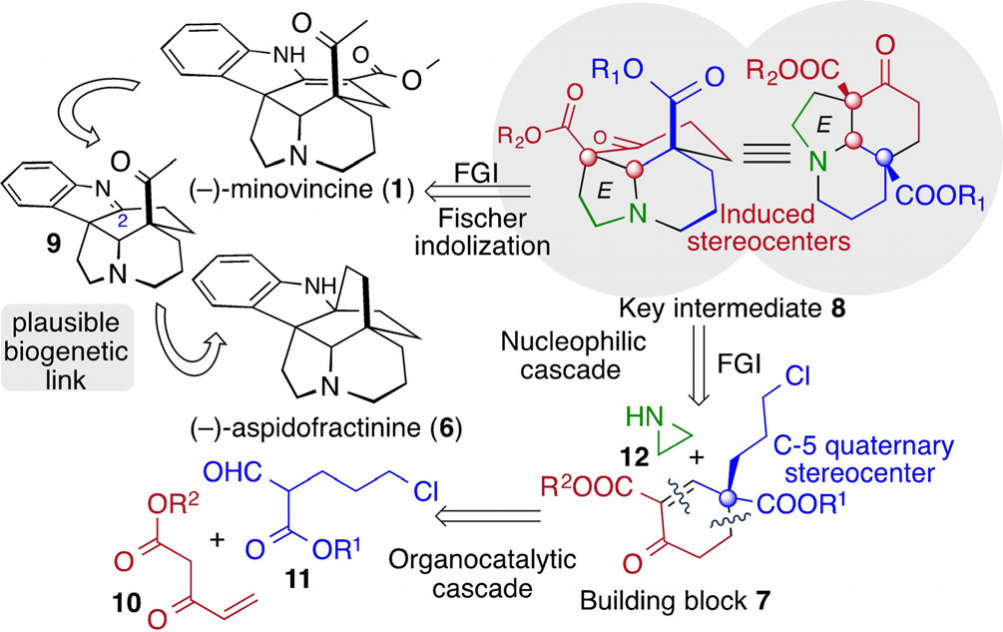
Retrosynthetic analysis of (−)‐minovincine (**1**) and (−)‐aspidofractinine (**6**).

As a first foray, we sought to develop an efficient synthesis of the desired enone **7** with the requisite quaternary stereogenic center. Specifically, we attempted to connect the easily available Nazarov reagent **10** and ω‐chloro‐formylpentenoate (**11**) using the previously reported quinine‐squaramide organocatalyst **13** 14 (Scheme 2). Gratifyingly, this Michael addition/aldol condensation organocascade reaction afforded the envisioned enone **7** as the sole product. By using optimized reaction conditions (dioxane, room temperature, 2 mol % catalyst), the chiral building block **7** was constructed in 71 % yield and 90 % *ee* (Scheme 2). Notably, the catalyst tolerated the presence of a primary alkyl chloride functional group; alkylative inhibition of the catalyst was not detected. This method proved also to be amenable for scale up, ultimately providing access to 80 g of enone **7**.

**Scheme 2:**
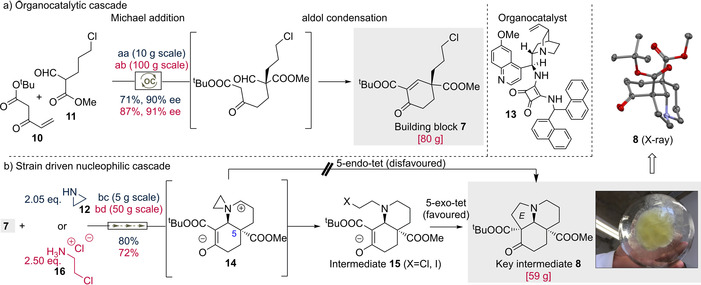
Rapid construction of key intermediate **8** through relay cascade reactions. Reaction conditions: aa) **10**+**11** (1.13:1.0) (2 mol % **13**) dioxane, r.t.; ab) **10**+**11** (1.1:1.0) (3 mol % **13**), dioxane, r.t.; ba) KI, CH_2_Cl_2_, rt.; bb) DIPEA, KI, CH_2_Cl_2_, r.t.. DIPEA=diisopropylethylamine.

With a robust approach to **7** in hand, we were poised to examine the feasibility of the nucleophilic cascade strategy toward the tricyclic ketone **8**. Pleasingly, the envisioned multistep reaction occurred smoothly and delivered the tricyclic ketone **8** with the correct configuration in high efficiency and diastereoselectivity (Scheme 2. **8**, X‐ray).^17^ Although the diastereoselectivity of the cascade was excellent, the exact mechanism by which this is achieved is not clear. To begin with, the product of the first aziridine adduct could not be detected by NMR, suggesting that the rate of the subsequent cyclization is relatively fast. Thus, the reaction started either by the aza‐Michael addition of aziridine (**12**) on the double activated enone **7** or by the nucleophilic substitution of aziridine (**12**) on the alkyl halide part of the enone **7**. Regardless of the reaction sequence, the stereochemical outcome is determined by the π‐facial diastereoselectivity of the aza‐Michael addition. This type of Michael addition/S_N_2 reaction or S_N_2reaction/Michael addition sequence thus establishes a preference for *trans* addition with the C‐5 carboxylic substituents of **14**.^18^ Since a 5‐*endo*‐tet cyclization is less probable,^19^ the presumed zwitterionic aziridinium intermediate **14** was expected to undergo a ring opening with halogenides. This mechanistic manifold was corroborated by the isolation of **15** from the reaction mixture.^20^ Once generated, the arising ethyl halogenide moiety was then quenched by the enolate, closing the cascade process and generating a quaternary stereogenic center and the E ring in parallel. As a less harmful and hazardous synthetic precursor of aziridine (**12**), 2‐chloroethylamine (**16**) was also tried. To our delight, the intriguing nucleophilic cascade also proceeded well with 2‐chloroethylamine (**16**) in the presence of DIPEA, which allowed us to conduct the process in a batch of 80 g with a 72 % yield.

Given the ready availability of tricyclic ketone **8**, the scalable synthesis of (−)‐minovincine (**1**) was addressed by using the classical Fischer indole synthesis^21^ (Scheme 3). The synthesis of indolenine **17** was straightforward, proceeded without incident. Thus, following selective deprotection of ^*t*^Bu‐ester, the resulting β‐oxo carboxylic acid spontaneously decarboxylated, affording ketone **18** in 95 % yield in 7 gram scale. Subsequent treatment of product **18** with phenylhydrazine generated aspidospermane‐type indolenine **17** and its structural isomer **19** in 50 % and 31 % yields, respectively. Having achieved the construction of pentacyclic **17**, what remained for completing the synthesis of minovincine was the installation of C‐3 methoxycarbonyl and C‐5 acetyl groups. During these endeavors, our priority was to minimize the functional group manipulations to shorten the synthetic route and enhance its practicality. First, methyl cyanoformate, also known as Mander's reagent, was used to append the methoxycarbonyl group into C‐3 position of the indolenine **20**. Importantly, some N‐carboxylated isomer **21** also formed that cannot be separated by chromatography (**20**/**21** ratio was 6:1). Then, we turned our attention to the regioselective transformation of the C‐5 methoxycarbonyl group to the requisite acetyl moiety. While conversion of the methyl ester **20** to a methyl ketone was not successful with standard reagents (MeLi⋅LiBr and TMSCH_2_MgCl), TMSCH_2_Li proved to be a competent reagent to effect the desired transformation^22^ Although our initial attempts with TMSCH_2_Li resulted in poor yields, we anticipated that the addition of TRIBAL to **20** would improve the selectivity of the reaction as a transient protecting group with dual roles. This strong base would not only exert a charge control over C‐3 methoxycarbonyl via N‐H deprotonation, but the diisobutyl aluminium adduct **22** would secure an enhanced steric shielding around the C‐3 methoxycarbonyl moiety.^20^ Gratifyingly, by employing TRIBAL additive, a 52 % yield (for two steps) of (−)‐minovincine (**1**) could be obtained on a 1.10 gram scale. Overall, the gram‐scale synthesis of (−)‐minovincine (**1**) was accomplished in an overall yield of 11 % by an eight‐step sequence. By virtue of (bio)synthetic potential of minovincine, this scalable route seems to provide rapid access toward structurally related, more complex indole alkaloids.

**Scheme 3:**
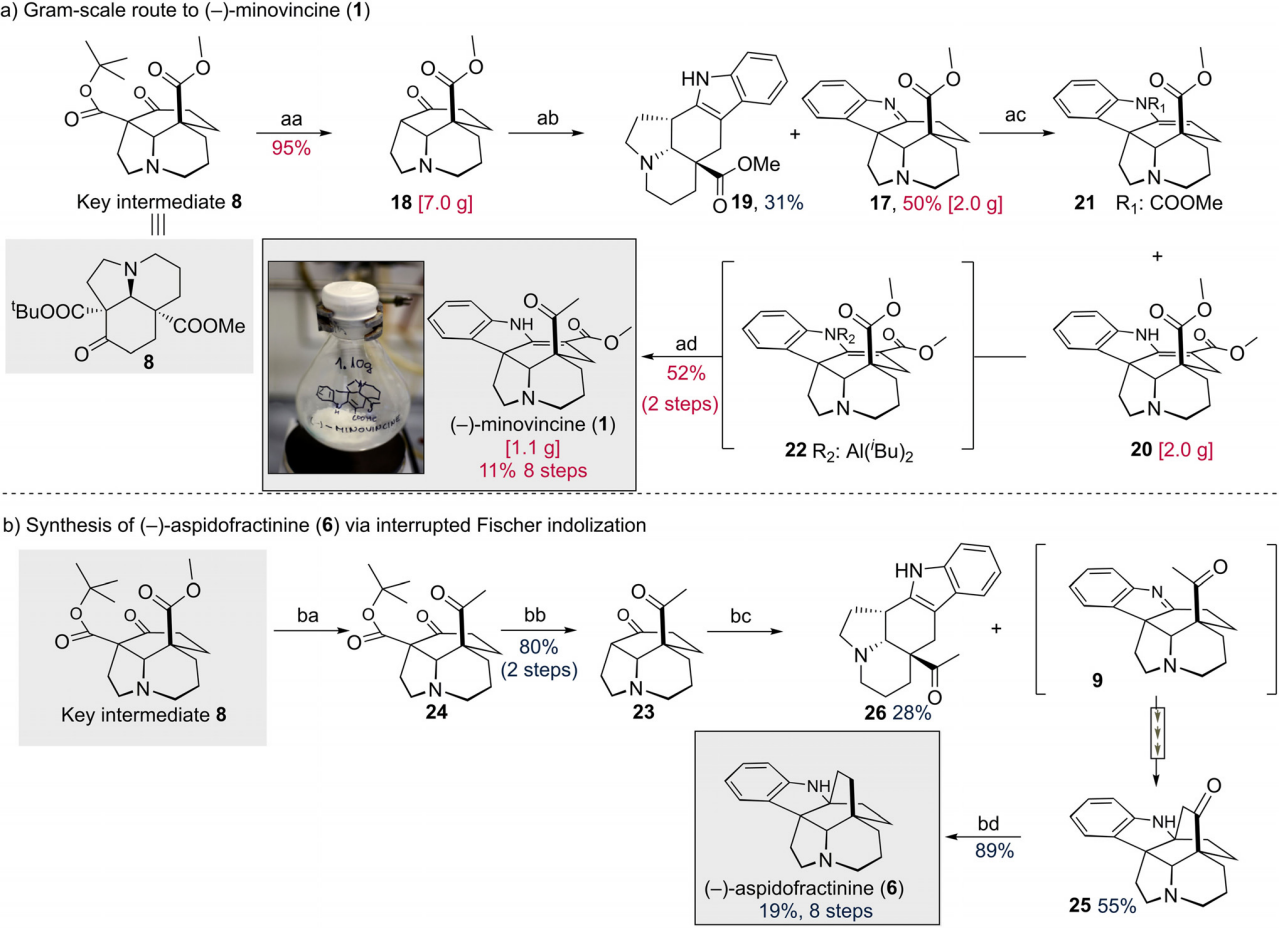
Syntheses of (−)‐minovincine (**1**) and (−)‐aspidofractinine (**6**). Reaction conditions: aa) aq. H_2_SO_4_ (50 V/V%), dioxane, r.t.; ab) PhNHNH_2_ then BF_3_⋅OEt_2_, MeOH, 70 °C; ac) ^*i*^Pr_2_NH, LDA, CNCOOMe, THF, −78 °C; ad) Al^*i*^Bu_3_, THF, −78 °C to r.t. then TMSCH_2_Li; ba) TMSCH_2_Li, THF, r.t.; bb) aq. H_2_SO_4_ (50 V V^−1^%), dioxane, r.t.; bc) PhNHNH_2_ then BF_3_⋅OEt_2_, EtOH, 85 °C; bd) N_2_H_4_⋅H_2_O, KOH, DEG, 130 to 210 °C, LDA=lithium diisopropylamide, TMS=trimethylsilyl, THF=tetrahydrofuran, DEG=diethyleneglycol.

Aspidofractinine is thought to originate from **9** that is derived from minovincine through ester hydrolysis followed by decarboxylation (Scheme 1).^1^, ^16^ While several routes have been developed to construct racemic aspidofractinine,^23^, ^24^ its only asymmetric synthesis was reported by Gagnon and Spino^25^ in 2009. The relative ease of our minovincine synthesis served as an impetus for developing a biosynthetically inspired synthesis of (−)‐aspidofractinine (**6**). Specifically, we envisioned a Fischer indolization^26^ interrupted by the pendant acetyl nucleophile. Therefore, we set out to prepare a C‐5 acetyl substituted tricyclic ketone **23** from the previously synthesized advanced intermediate **8**. We reasoned that the steric hindrance embedded in this key intermediate **8** can be exploited for chemo‐ and regioselective transformation, thus, obviating the need of protecting group manipulation. This scenario proved to be viable, affording a simple route to **24**. Thus, treatment of **8** with TMSCH_2_Li (rt, 1 h) followed by ester hydrolysis and decarboxylation resulted in the selective generation of **23** with 80 % overall yield. Corroborating the importance of steric effect, the decarboxylated and thus sterically less crowded analogue **18** was also reacted with TMSCH_2_Li under the same conditions. Importantly, that reaction resulted in a complex mixture.^20^

Next, the feasibility of the interrupted Fischer indolization was investigated to construct the cage‐like aspidofractinine framework. To our delight, the Fischer indole/Mannich cascade reaction occurred smoothly to afford the corresponding oxo‐aspidofractinine **25** in a 55 % yield (alongside with its isomer **26**) via presumed indolenine intermediate **9**. Several significant features of the reactions shown in Scheme 3 should be noted. Although there are scattered examples of interrupted Fischer indolization in the literature, a Mannich reaction coupled strategy has not been utilized, to best of our knowledge. Furthermore, the successful implementation of the *trans* annular Mannich reaction/Fischer indolization process led to the formation of three new bonds and two additional quaternary stereogenic centers. Moreover, based on the notion that C‐20‐derived indole formation was not detected, we surmised that the steric effect secured again the regioselectivity. As the final step of the synthetic route, the substrate **25** was exposed to hydrazine to furnish (−)‐aspidofractinine (**6**) in 89 % yield.

In conclusion, we have developed eight‐step synthetic routes toward (−)‐minovincine (**1**) and (−)‐aspidofractinine (**6**) with 11 % and 19 % overall yields, respectively. Key to the success was the strategic implementation of a chain of cascade reactions, including organocatalytic Michael addition/aldol condensation, multistep anionic Michael/S_N_2 cascade reaction, and Mannich reaction interrupted Fischer indolization. Importantly, four contiguous stereogenic centers were created in those steps with excellent absolute and relative stereochemical control. Both the employed sequence of cascade reactions and the steric‐effect‐steered chemo‐ and regioselective reactions contributed substantially to achieving synthetic brevity but also excellent practicality. Thus, the advanced building block **8**, which has a quaternary stereogenic center, could be synthesized on a 60 g scale. (−)‐minovincine (**1**) was delivered on a gram scale and the cage‐like (−)‐aspidofractinine (**6**) was accessible via an impressively short sequence. Furthermore, the use of easily available, inexpensive reagents adds further synthetic convenience. We are currently pursuing this synthetic strategy in the total synthesis of structurally related members of aspidospermanes.

## Conflict of interest

The authors declare no conflict of interest.

## Supporting information

As a service to our authors and readers, this journal provides supporting information supplied by the authors. Such materials are peer reviewed and may be re‐organized for online delivery, but are not copy‐edited or typeset. Technical support issues arising from supporting information (other than missing files) should be addressed to the authors.

Supplementary
