# Supplementary material for: Total Syntheses of (−)‐Minovincine and (−)‐Aspidofractinine through a Sequence of Cascade Reactions
Source: Angew Chem Int Ed Engl. 2020 Jun 3;59(32):13547–51. doi: 10.1002/anie.202004769 (PMC7497198; doi:10.1002/anie.202004769)
Supplement: Supplementary file 1 — Supplementary [file ANIE-59-13547-s001.pdf]

Angewandte Chemie
Eine Zeitschrift der Gesellschaft Deutscher Chemiker

Supporting Information

Total Syntheses of (−)-Minovincine and (−)-Aspidofractinine through a Sequence of Cascade Reactions

*Szilárd Varga, Péter Angyal, Gábor Martin, Orsolya Egyed, Tamás Holczbauer, and Tibor Soós**

anie_202004769_sm_miscellaneous_information.pdf

CONTENTS

General information S3

Experimental procedures and characterisations S4

Scalable synthesis of starting materials S4

Synthesis of *tert*-butyl 3-oxopent-4-enoate 10 ..... S4

Synthesis of methyl 5-chloro-2-formylpentanoate 11 ..... S6

Scalable synthesis of tricyclic key intermediate S7

Synthesis of building block 7 ..... S7

Synthesis of tricycle 8 ..... S9

Functional group interconversions towards Minovincine S12

Synthesis of ketone 18 ..... S12

Synthesis of indolenine 17 ..... S14

Synthesis of diester 20 ..... S18

Synthesis of (-)-minovincine (1) S21

Functional group interconversions towards Aspidofractinine S24

Synthesis of diketone 23 ..... S24

Synthesis of oxo-aspidofractinine 25 ..... S27

Synthesis of (-)-aspidofractinine (6) S32

Further experimental results S34

Optimization of the organocatalytic cascade S34

Optimization of the anionic cascade S35

Isolation and NMR studies on the formation of Intermediate 15 ..... S36

Synthesis of diketone 23 via ketone 18 - experimental proof of steric shielding S38

Optimization of the last step towards (-)-minovincine S39

NMR studies on the formation of adduct 22 ..... S40

Chiral HPLC data – building block 7 ..... S41

X-ray data – tricycle 8 ..... S42

GENERAL INFORMATION

All reactions were carried out using oven-dried glassware and anhydrous solvents unless noted otherwise. Flash silica chromatography was performed on silica gel (ZEOprep 60 25-40 μm, ZEOCHEM) with the indicated eluents. Thin-layer chromatography was performed on silica plates (Kieselgel 60 F_{254} Merck). Compounds were visualized by UV (254 nm) or Ceric Ammonium Molybdate (CAM) staining. HRMS Spectra were obtained using Waters Q-TOF Premier Mass Spectrometer (Waters Co. Milford, MA USA), using electrospray ionization (ESI). ^{1}H and ^{13}C NMR spectra were recorded using a Varian 500 MHz spectrometer. Chemical shifts are referenced to the residual solvent signals ($CHCl_3$: δ = 7.26 ppm for ^{1}H, δ = 77.0 ppm for ^{13}C). Data are reported as follows: chemical shifts (ppm), multiplicity (s = singlet, d = doublet, t = triplet, q = quartet, br = broad, m = multiplet), coupling constants (Hz), and integration. Resonance assignments are based on standard homo- (gDQCOSY, 2D TOCSY, 2D NOESY) and hetero-correlation (^{1}H-^{13}C gHSQCAD, ^{1}H-^{13}C gHMBCAD) measurements, performed at 600 MHz on a Varian VNMR SYSTEM™ using a triple resonance probe equipped with Z pulse field gradient. All spectra were recorded with the standard spectrometer pulse sequences and settings. The enantiomeric excess of the products was determined on a chiral stationery phase HPLC (Daicel Chiralpak IC 250x4.6 mm, 5 μm column). Optical Rotation was determined using a JASCO P-2000 polarimeter.

All starting materials were purchased from Aldrich or TCI and used without further purification unless stated otherwise. Anhydrous THF and dioxane were distilled from sodium/benzophenone, while DCM from calcium hydride.

Experimental procedures and characterisations

Scalable synthesis of starting materials

Synthesis of *tert*-butyl 3-oxopent-4-enoate 10

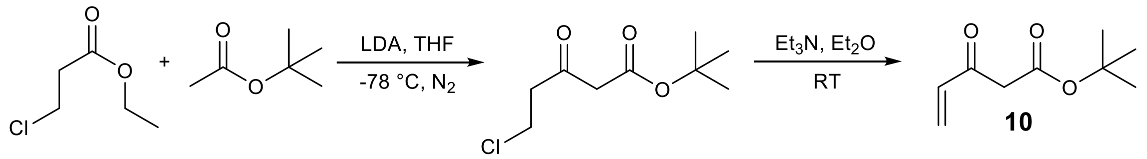

Multigram-scale version of previously reported procedure[1]

Solution A
A flame dried three-necked 500 mL round bottom flask equipped with a magnetic stirring bar was charged with anhydrous THF (240 mL) under nitrogen atmosphere. Diisopropylamine (38.0 mL, 0.272 mol, 1.36 eq.) was added via syringe and the solution was cooled to -78 °C. *n*-Butyllithium in hexanes (111.0 mL, 2.45 M, 1.36 eq.) was added via syringe in three portions (2 x 50.0 mL then 11.0 mL) and the solution was stirred for 30 minutes at -78 °C. Then *tert*-Butylacetate (38.0 mL, 0.284 mol, 1.42 eq.) was added via syringe over 10 minutes and the solution was stirred for 1 h at -78 °C, meanwhile Solution B was prepared.

Solution B
A flame dried three-necked 1000 mL round bottom flask equipped with a magnetic stirring bar was charged with anhydrous THF (280 mL) under nitrogen atmosphere. Ethyl 3-chloropropanoate (25.3 mL, 0.200 mol, 1.00 eq.) was added via syringe and the solution was cooled to -78 °C.

After preparing the two solutions, Solution A was added to Solution B via a cannula using nitrogen overpressure over 30 minutes. The resulting solution was stirred for an additional 15 minutes at -78 °C, then quenched with glacial acetic acid (120 mL) while maintaining the temperature at -78 °C. The cooling bath was removed and the suspension was left to warm to RT. Diethyl ether (1000 mL) and distilled water (400 mL) was added and the phases were separated. The organic phase was washed with aq. K_2CO_3 (400 ml, 20 m/m%) and brine (200 mL) and dried over Na_2SO_4. After filtration Et_3N was added (35.0 mL, 0.252 mol, 1.26 eq.), as a result the solution became opaque. The mixture was stirred for 16 h at RT, while the round bottom flask became covered with white crystals of $Et_3N{\cdot}HCl$. After filtration

[1] S. Ohta, A. Shimabayashi, S. Hatano, M. Okamoto, *Synthesis* **1983**, 715–716

the organic phase was washed with aq. HCl (240 mL, 5 m/m%) and brine (240 mL), dried over Na_2SO_4, filtered and evaporated under reduced pressure using a water bath heated to 30 °C. A yellowish crude oil was obtained (38–40 g). Catalytic amount of BHT (100 mg, 0.23 mol%) was added and the product was purified by vacuum distillation (0.1 mbar, 60–105 °C oil bath temperature). The desired product **5** was obtained as a transparent oil (25.5 g, 75% yield) and stored in freezer after the addition of BHT (50 mg).

All analytical data were in accordance with data reported in the literature.[1]

Synthesis of methyl 5-chloro-2-formylpentanoate 11

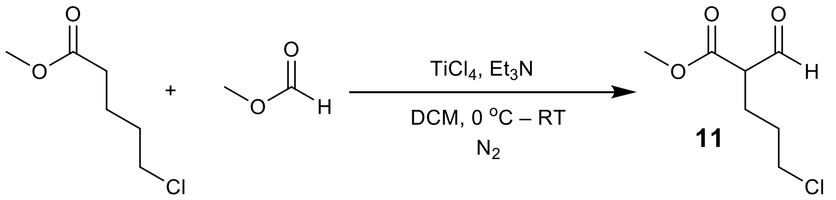

Multigram-scale version of previously developed procedure[2]

A flame dried three-necked round bottom flask equipped with a magnetic stirring bar and thermometer was charged with anhydrous DCM (330 mL) under nitrogen atmosphere. Methyl 5-chloropentanoate (25.0 g, 0.166 mol, 1.00 eq.) and methyl formate (30.7 mL, 0.500 mol, 3.0 eq.) was added and the resulting mixture was cooled to 0 °C. $TiCl_4$ (36.5 mL, 0.332 mol, 2.0 eq.) was added dropwise while maintaining the internal temperature below 5 °C. Then Et_3N (55.6 mL, 0.400 mol, 2.4 eq.) was added dropwise, again keeping the temperature below 5 °C. The mixture was stirred at 0 °C for 1 h, then left to warm to room temperature and stirred for further 1 h. The solution was then treated with distilled water (200 mL) and extracted with EtOAc (3 x 200 mL). The combined organic extracts were washed with brine (200 mL), dried over Na_2SO_4 and concentrated. The resulting crude product (32.2 g) was purified by vacuum distillation (bp.: 65–73 °C/ 2 mmHg). The desired product **11** was obtained as a transparent oil (28.0 g, 94% yield).

All analytical data were in accordance with data reported in the literature.[2]

[2] H. Nakatsuji, H. Nishikado, K. Ueno, Y. Tanabe, *Org. Lett.* **2009**, *11*, 4258–4261

SCALABLE SYNTHESIS OF TRICYCLIC KEY INTERMEDIATE

Synthesis of building block 7

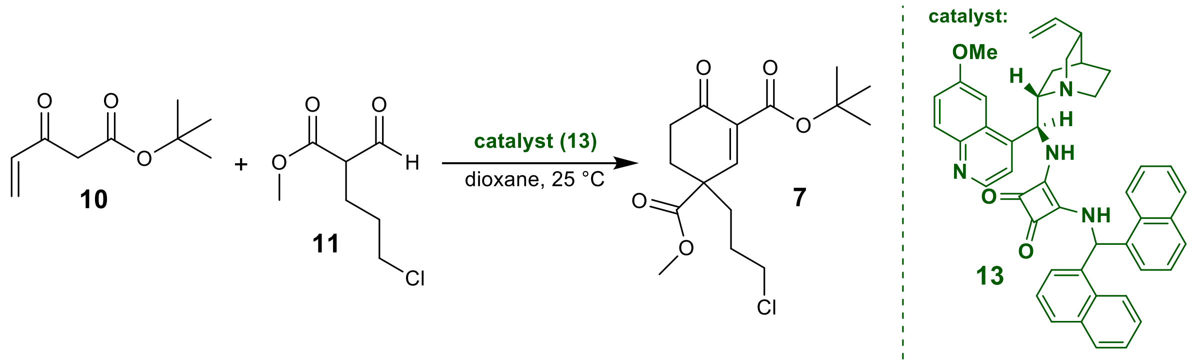

Small-scale procedure:

To a solution of **11** (12.0 g, 67.0 mmol, 1.00 eq.) and **10** (12.7 g, 76.0 mmol, 1.13 eq.) in dioxane (70 mL) was added **13** (890 mg, 1.30 mmol, 2 mol%). The mixture was stirred vigorously at room temperature for 6 days and then concentrated *in vacuo*. The crude product was purified by flash column chromatography on flash silica gel (eluted with hexanes/ethyl acetate 3:1–1:1) to afford the desired product **7** as a pale yellow oil (15.7 g, 71% yield).

Large-scale procedure

To a solution of **11** (50.0 g, 280.0 mmol, 1.00 eq.) and **10** (50.0 g, 300.0 mmol, 1.1 eq.) in dioxane (280 mL) was added **13** (5.75 g, 8.40 mmol, 3 mol%). The mixture was stirred vigorously at room temperature for 6 days and then concentrated *in vacuo*. The crude product was purified by flash column chromatography on flash silica gel (eluted with hexanes/ethyl acetate 3:1–1:1) to afford the desired product **7** as a pale yellow oil (80.27 g, 87% yield).

R_f = 0.45 (hexanes/ethyl acetate 3:1) [$KMnO_4$].

$[\alpha]_D^{23}$ = +5.3 (c = 1.0; $CHCl_3$).

HRMS (ESI): calcd. for $C_{16}H_{23}ClNaO_5$ $[M+Na]^+$ 353.1132, found 353.1129.

^{1}H NMR ($CDCl_3$, 500 MHz, δ, ppm): 7.33 (1H, s); 3.76 (3H, s); 3.52 (2H, m); 2.51 (2H, m); 2.44 (1H, m); 1.98 (2H, m); 1.90 (1H, m); 1.77 (2H, m); 1.50 (9H, s).

^{13}C NMR ($CDCl_3$, 125 MHz, δ, ppm): 193.2; 172.5; 163.5; 152.4; 133.8; 82.2; 52.7; 47.4; 44.2; 35.6; 35.2; 29.7; 28.0 (3C); 27.5.

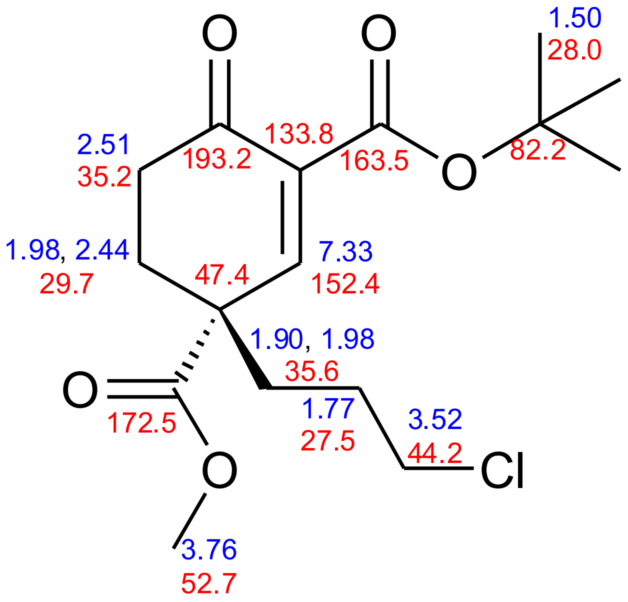
O
O
1.50
28.0
2.51
35.2
193.2
133.8
163.5
O
82.2
1.98, 2.44
29.7
47.4
7.33
152.4
1.90, 1.98
35.6
O
172.5
1.77
27.5
3.52
44.2
Cl
O
3.76
52.7

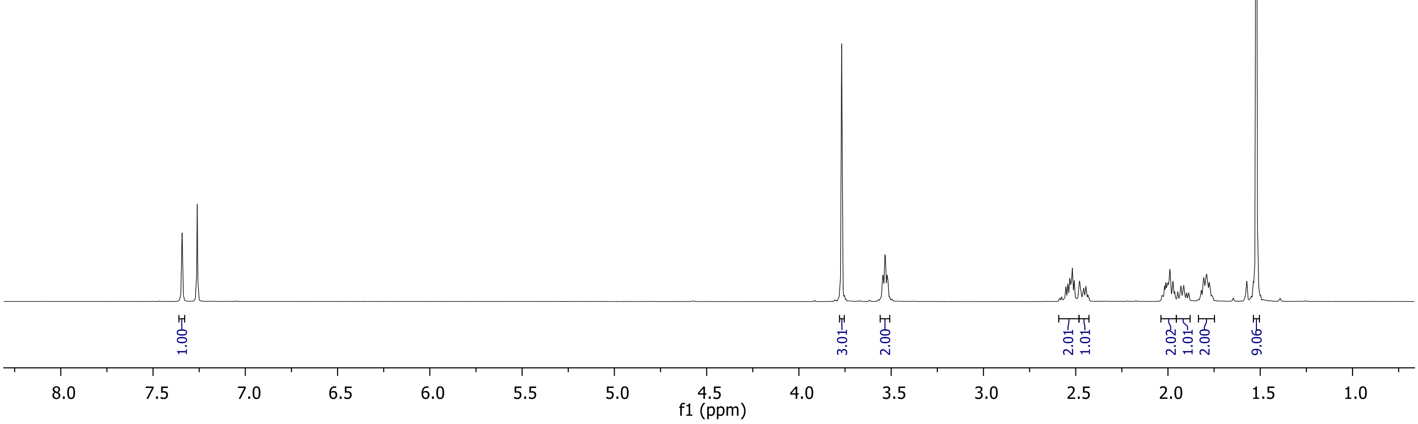
1.00
3.01
2.00
2.01
1.01
2.02
1.01
2.00
9.06
8.0
7.5
7.0
6.5
6.0
5.5
5.0
4.5
4.0
3.5
3.0
2.5
2.0
1.5
1.0
f1 (ppm)

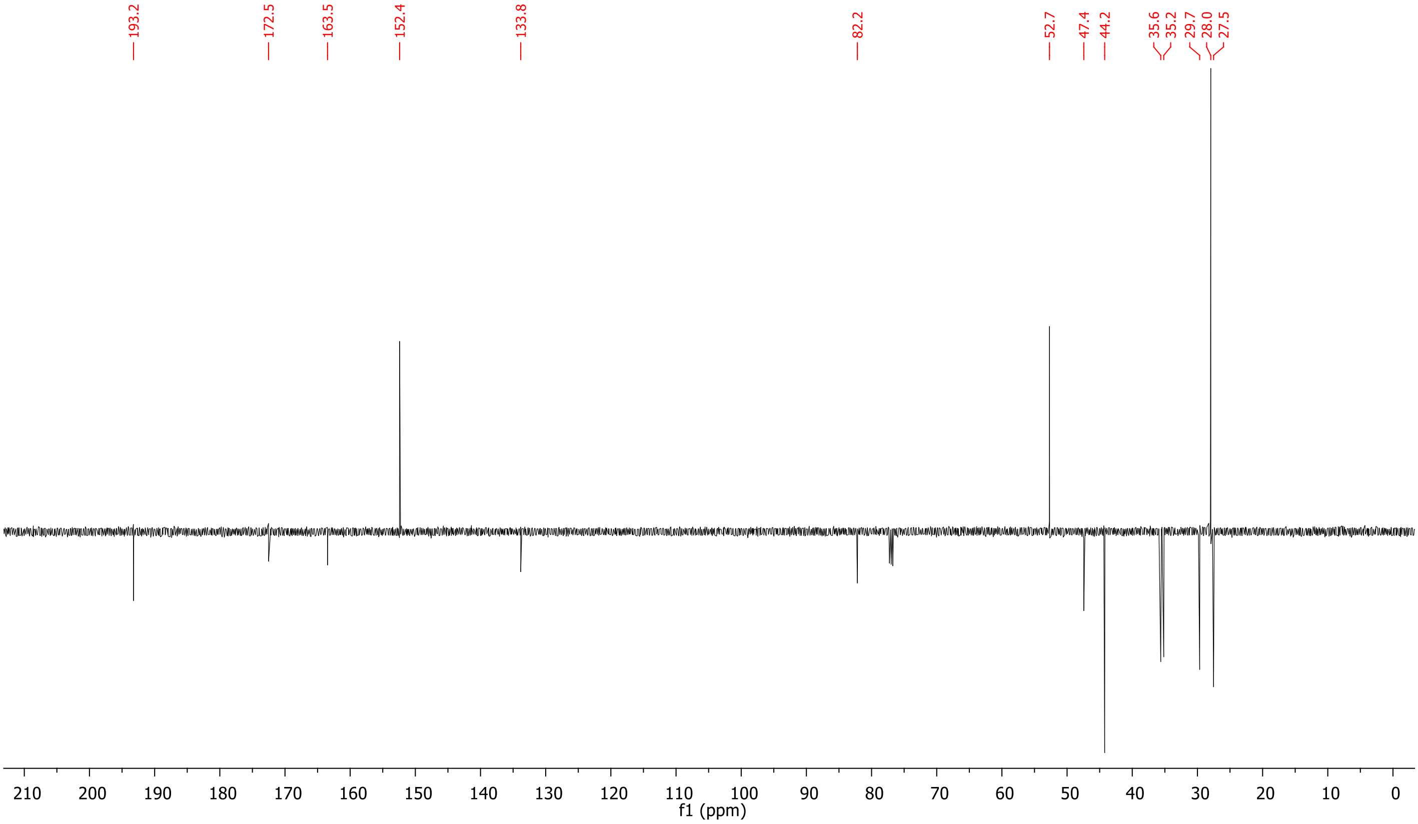
193.2
172.5
163.5
152.4
133.8
82.2
52.7
47.4
44.2
35.6
35.2
29.7
28.0
27.5
210
200
190
180
170
160
150
140
130
120
110
100
90
80
70
60
50
40
30
20
10
0
f1 (ppm)

Synthesis of tricycle 8

Small-scale procedure with aziridine

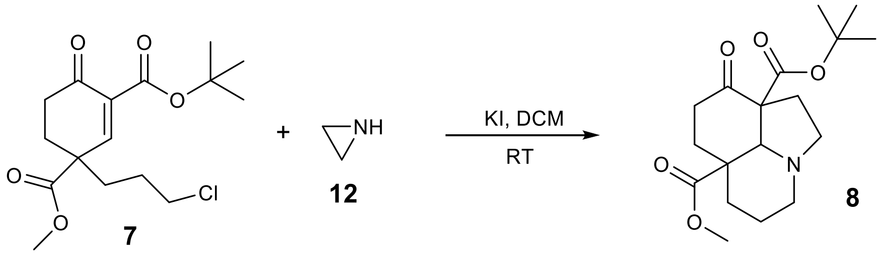

A flame dried three-necked round bottom flask equipped with a magnetic stirring bar was charged with anhydrous DCM (24.0 mL) under nitrogen atmosphere. Enone **7** (2.00 g, 6.05 mmol, 1.00 eq.) was dissolved in the solvent. Anhydrous KI (1.054 g, 6.35 mmol, 1.05 eq.) and aziridine (641 µL, 12.4 mmol, 2.05 eq.) were added under nitrogen atmosphere and the resulting suspension was stirred for 16 h at room temperature. The solvent was concentrated to ~5 mL and purified by flash column chromatography directly (eluted with hexanes/ethyl acetate 5:1). The product **8** was obtained as a yellow oil, which crystallised upon cooling and standing at room temperature (1.63 g, 80% yield).

Large-scale procedure with 2-chloroethylamine hydrochloride

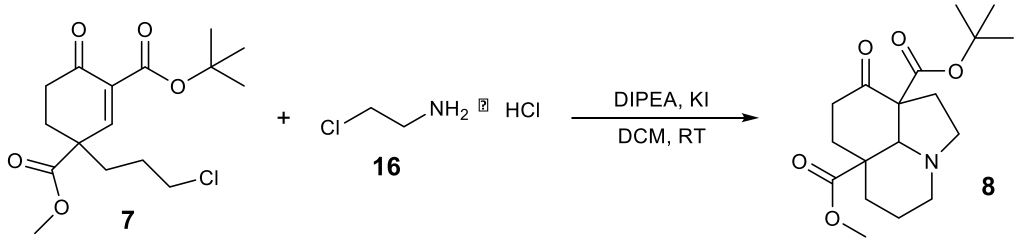

A flame dried three-necked round bottom flask equipped with a mechanical stirrer was charged with anhydrous DCM (1200 mL) under nitrogen atmosphere. Enone **7** (80.27 g, 243 mmol, 1.00 equiv.) was dissolved in the solvent. Anhydrous KI (44.4 g, 267 mmol, 1.10 eq.), 2-chloroethylamine hydrochloride (70.47 g, 608 mmol, 2.50 eq.) and *N,N*-diisopropylethylamine (310 mL, 1.82 mol, 7.50 eq.) were added under nitrogen atmosphere and the resulting suspension was stirred for 16 h at room temperature. The crude reaction mixture was concentrated *in vacuo*, the resulting residue was dissolved in DCM (700 mL) and treated with aq. Na_2CO_3 (prepared by mixing 700 mL distillated water + 700 mL sat. aq. Na_2CO_3). After separating the phases, the organic layer was washed three more times with aq. Na_2CO_3 (prepared by mixing 200 mL distillated water + 200 mL sat. aq. Na_2CO_3) and dried over Na_2SO_4. The solvent was evaporated *in vacuo* and the residue was triturated with hexanes (250 mL). The precipitate was filtered and the mother liquor was concentrated *in*

vacuo. The residue was purified by flash column chromatography (eluted with hexanes/ethyl acetate 5:1). The product **8** was obtained as a yellow oil, which crystallised upon cooling and standing at room temperature (58.7 g, 72% yield).

R_f = 0.50 (hexanes/ethyl acetate 3:1) [$KMnO_4$].

$[\alpha]_D^{23}$ = -55.8 (c = 1.0; $CHCl_3$).

HRMS (ESI): calcd. for $C_{18}H_{28}NO_5$ $[M+H]^+$ 338.1967, found 338.1958.

^{1}H NMR ($CDCl_3$, 500 MHz, δ, ppm): 3.74 (3H, s); 3.09 (1H, s); 3.01 (1H, ddd, *J* = 9.1, 8.3, 2.9 Hz); 3.00 (1H, m); 2.90 (1H, ddd, *J* = 14.4, 14.2, 6.2 Hz); 2.77 (1H, ddd, *J* = 13.1, 9.1, 7.8 Hz); 2.42 (1H, ddd, *J* = 14.4, 4.0, 2.5 Hz); 2.37 (1H, ddd, *J* = 14.2, 13.5, 4.0 Hz); 2.24 (1H, ddd, *J* = 10.2, 8.3, 7.8 Hz); 2.07 (1H, ddd, *J* = 13.5, 6.2, 2.5 Hz); 2.03 (1H, td, *J* = 11.5, 2.6 Hz); 1.92 (1H, ddd, *J* = 13.1, 10.2, 2.9 Hz); 1.74 (2H, m); 1.70 (1H, m); 1.53 (1H, m); 1.41 (9H, s).

^{13}C NMR ($CDCl_3$, 125 MHz, δ, ppm): 205.8; 175.5; 170.5; 82.2; 71.5; 64.2; 52.4; 52.2; 52.0; 45.5; 38.0; 33.1; 29.2; 27.8 (3C); 27.1; 20.7.

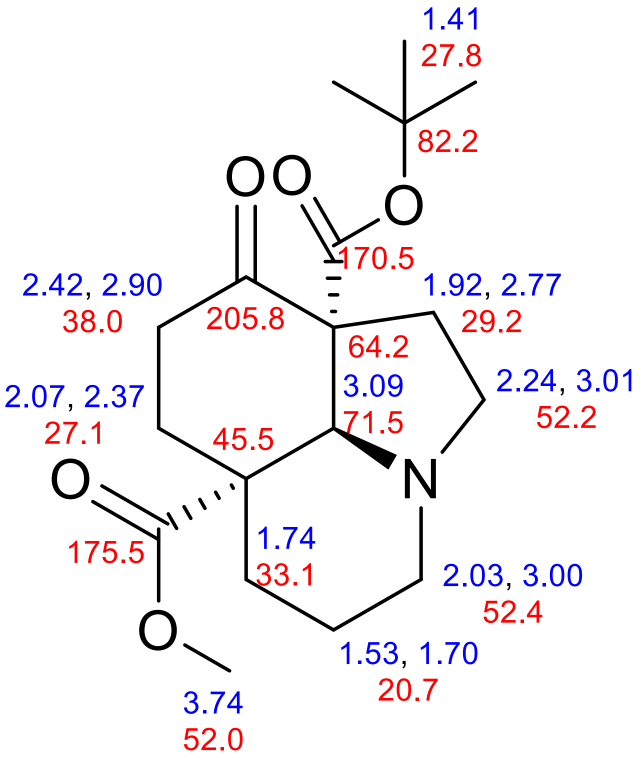
1.41
27.8
82.2
O
O
O
170.5
1.92, 2.77
29.2
2.42, 2.90
38.0
205.8
64.2
3.09
71.5
2.24, 3.01
52.2
2.07, 2.37
27.1
45.5
N
O
175.5
1.74
33.1
2.03, 3.00
52.4
O
1.53, 1.70
20.7
3.74
52.0

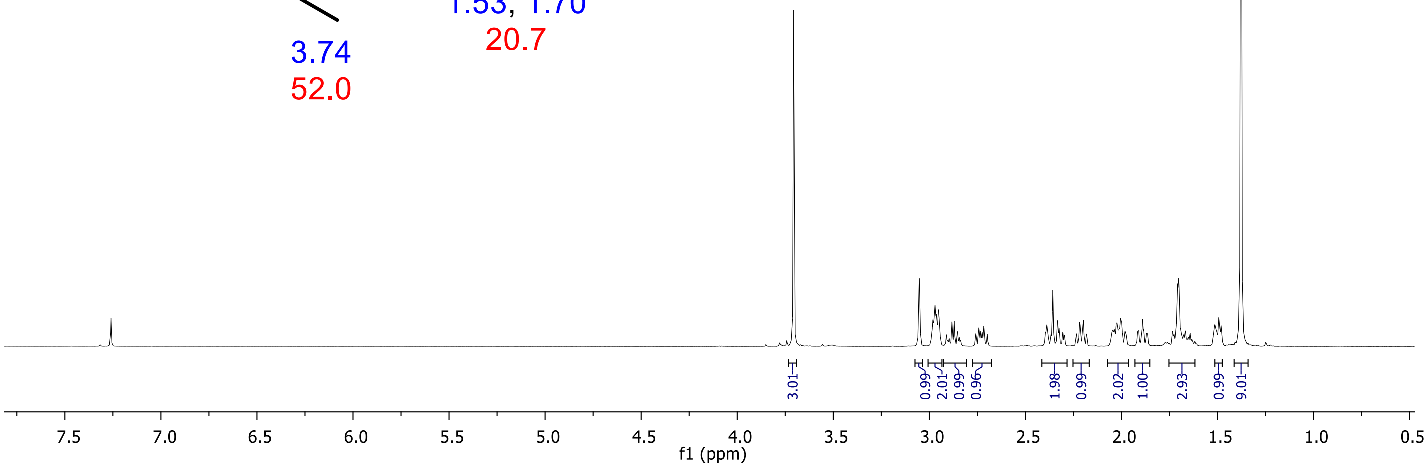
3.01
0.99
2.01
0.99
0.96
1.98
0.99
2.02
1.00
2.93
0.99
9.01
7.5
7.0
6.5
6.0
5.5
5.0
4.5
4.0
3.5
3.0
2.5
2.0
1.5
1.0
0.5
f1 (ppm)

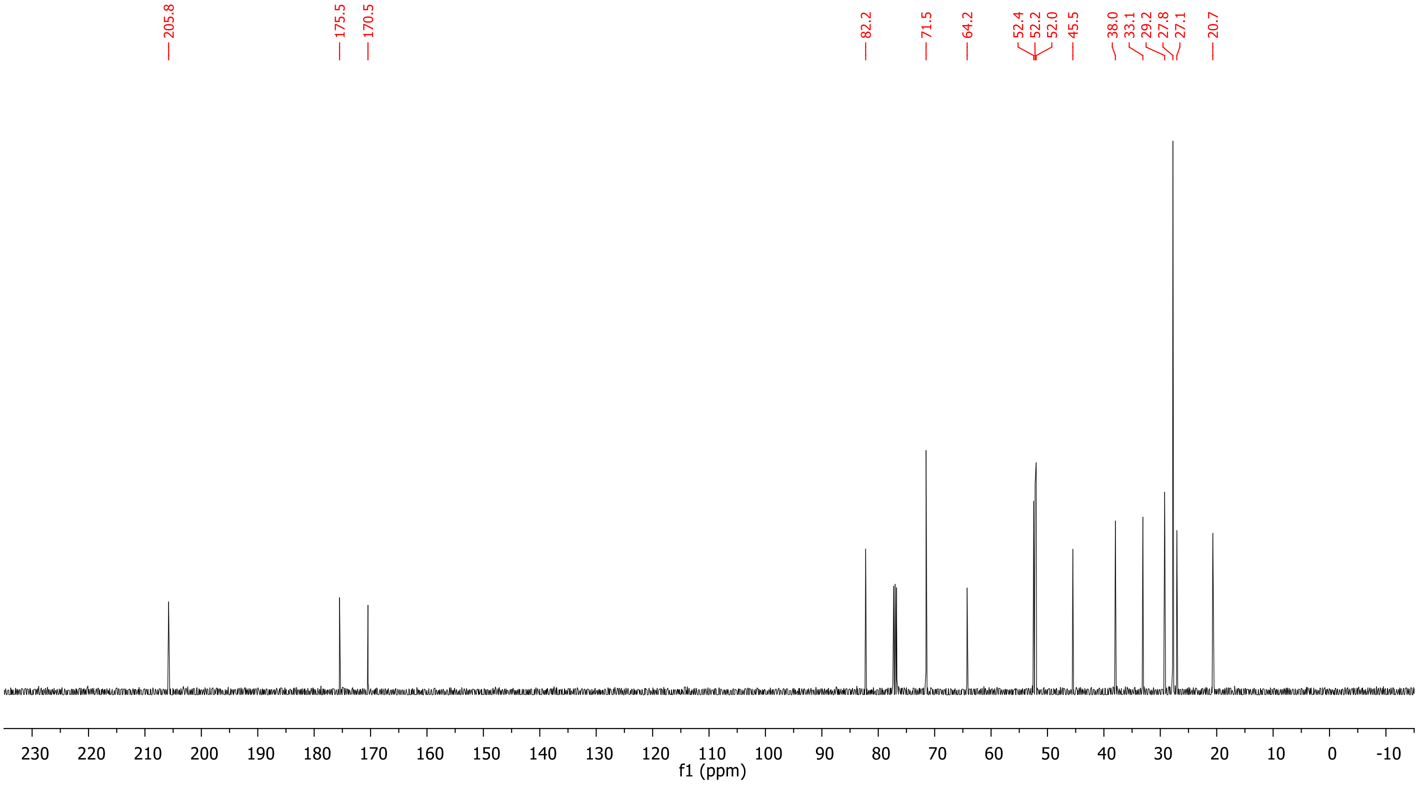
205.8
175.5
170.5
82.2
71.5
64.2
52.4
52.2
52.0
45.5
38.0
33.1
29.2
27.8
27.1
20.7
230
220
210
200
190
180
170
160
150
140
130
120
110
100
90
80
70
60
50
40
30
20
10
0
-10
f1 (ppm)

FUNCTIONAL GROUP INTERCONVERSIONS TOWARDS MINOVINCINE

Synthesis of ketone 18

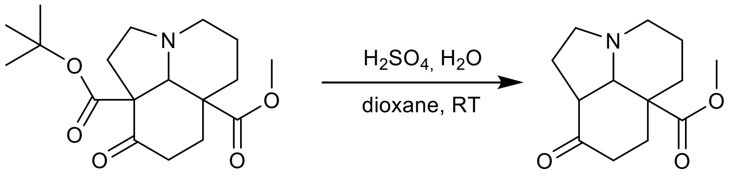

Tricycle **8** (10.29 g, 30.44 mmol, 1.00 eq.) was dissolved in dioxane (80 mL) and aq. H_2SO_4 (80 mL, 50 V/V%) was added slowly (~20 min), while keeping the temperature below 15 °C with an ice/water cooling bath. The solution was stirred for further 2 h at room temperature. The solution was then treated with sat. aq. Na_2CO_3 (keeping the temperature below 15 °C again) until reaching pH = 10 (NOTE: harsh gas evolution was observed) and extracted with DCM (3 x 80 mL). The combined organic extracts were dried over Na_2SO_4 and concentrated. A brown crude oil was obtained which was purified by flash column chromatography on flash silica gel (eluted with hexanes/ethyl acetate 2:1) to afford the desired product **18** as a yellow oil (6.87 g, 95% yield).

R_f = 0.50 (hexanes/ethyl acetate 2:1) [$KMnO_4$].

$[\alpha]_D^{23}$= +29.1 (c = 1.0; $CHCl_3$).

HRMS (ESI): calcd. for $C_{13}H_{20}NO_3$ $[M+H]^+$ 238.1443, found 238.1440.

^{1}H NMR ($CDCl_3$, 500 MHz, δ, ppm): 3.78 (3H, s); 3.04 (1H, td, *J* = 9.3, 3.3 Hz); 3.01 (1H, m); 2.88 (1H, ddd, *J* = 9.5, 5.2, 2.1 Hz); 2.62 (1H, d, *J* = 5.2 Hz); 2.50 – 2.33 (3H, m); 2.25 (1H, ddd, *J* = 14.8, 14.4, 5.3 Hz); 2.02 (2H, m); 1.91 (1H, ddd, *J* = 10.9, 3.8, 1.5 Hz); 1.88 (1H, ddd, *J* = 13.8, 9.4, 2.8 Hz); 1.80 – 1.70 (2H, m); 1.57 (1H, m); 1.51 (1H, m).

^{13}C NMR ($CDCl_3$, 125 MHz, δ, ppm): 209.9; 176.2; 67.8; 52.5; 52.4; 52.0; 49.1; 45.6; 38.3; 32.3; 27.0; 21.1; 21.0.

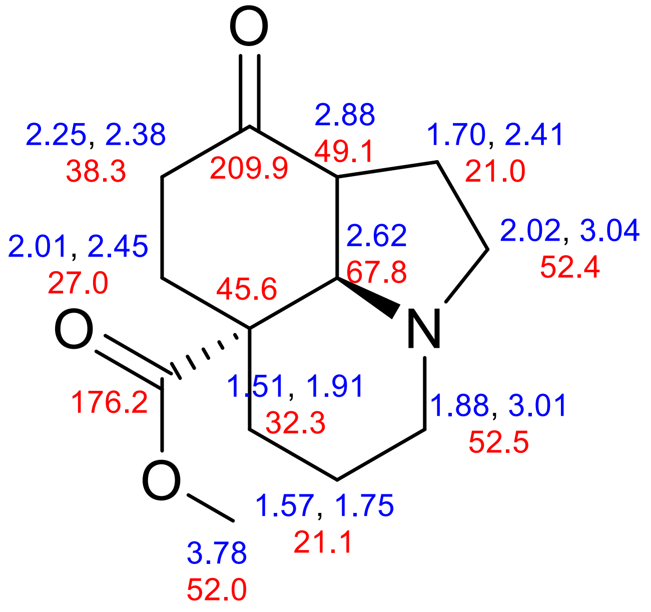
O
2.25, 2.38
38.3
209.9
2.88
49.1
1.70, 2.41
21.0
2.01, 2.45
27.0
2.62
67.8
2.02, 3.04
52.4
45.6
O
N
176.2
1.51, 1.91
32.3
1.88, 3.01
52.5
O
1.57, 1.75
21.1
3.78
52.0

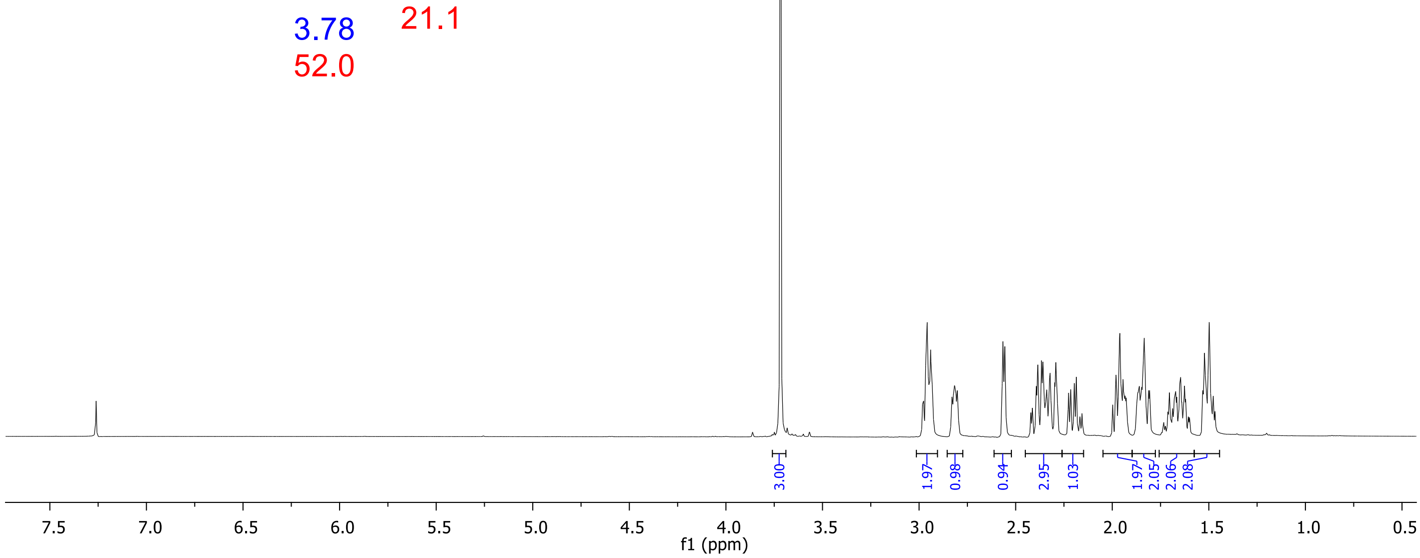
3.00
1.97
0.98
0.94
2.95
1.03
1.97
2.05
2.06
2.08
7.5 7.0 6.5 6.0 5.5 5.0 4.5 4.0 3.5 3.0 2.5 2.0 1.5 1.0 0.5
f1 (ppm)

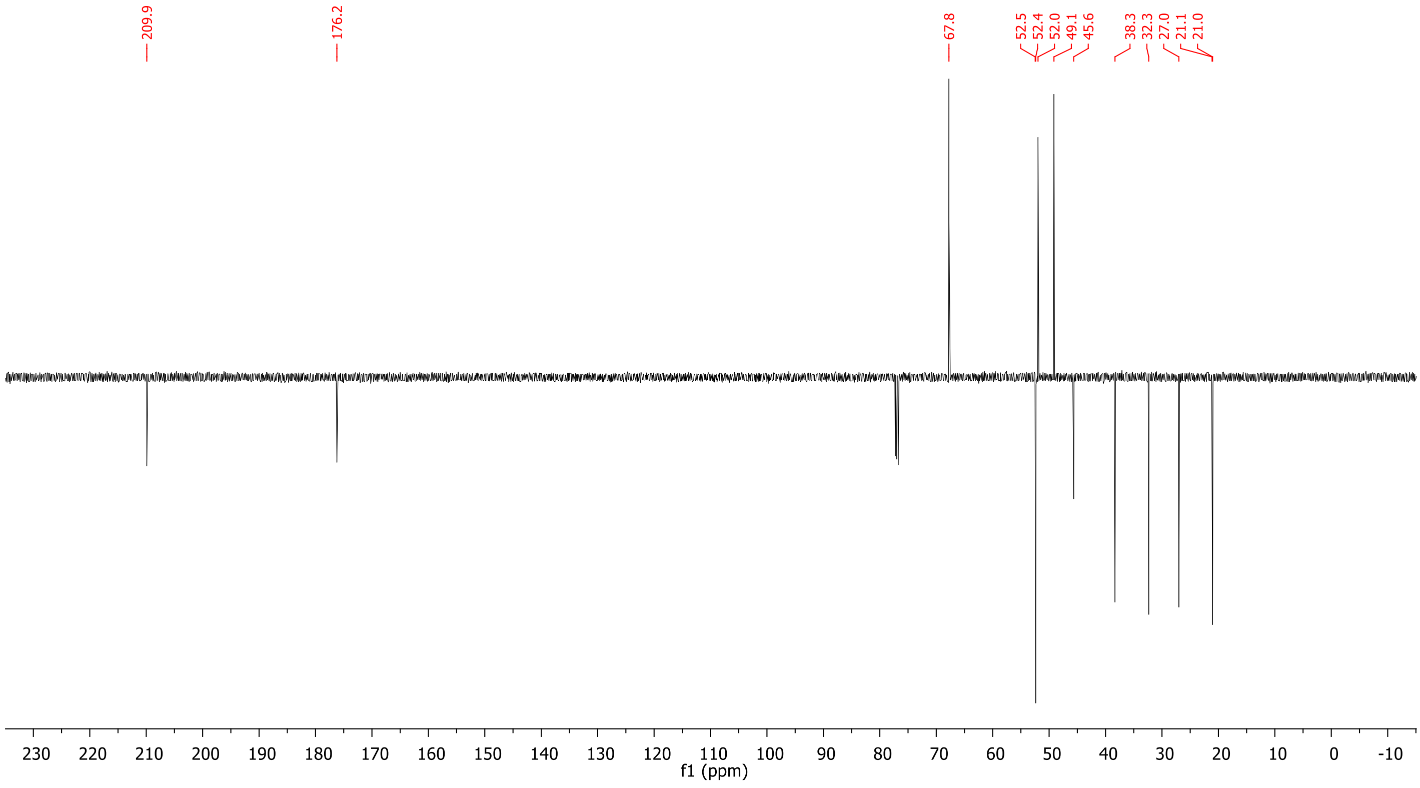
209.9
176.2
67.8
52.5
52.4
52.0
49.1
45.6
38.3
32.3
27.0
21.1
21.0
230 220 210 200 190 180 170 160 150 140 130 120 110 100 90 80 70 60 50 40 30 20 10 0 -10
f1 (ppm)

Synthesis of indolenine 17

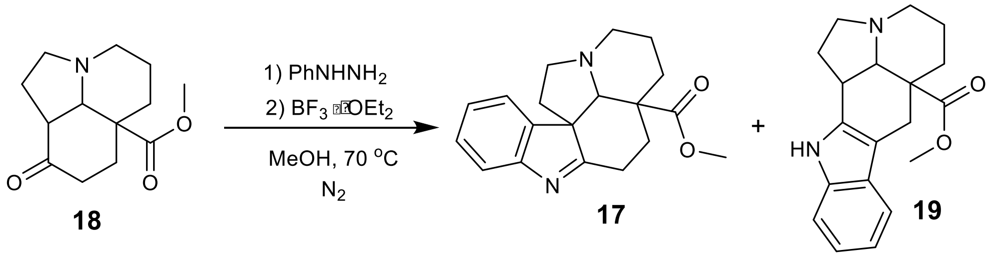

To a solution of ketone **18** (3.03 g, 0.013 mol, 1.00 eq.) in MeOH (40.0 mL) in a sealed tube was added $PhNHNH_2$ (1.34 mL, 0.014 mol, 1.08 eq.). The reaction mixture was placed in a preheated oil bath at 70 °C and stirred for 2 h under N_2 atmosphere. The reaction mixture was cooled to room temperature and $BF_3·OEt_2$-MeOH solution (60.0 mL, 1:9 V/V) was added in one portion. The resulting mixture was placed in a preheated oil bath and stirred at 70 °C for 16 h. The resulting brown solution was cooled to room temperature, treated with sat. aq. Na_2CO_3 (100 mL) and extracted with DCM (3 x 100 mL). The combined organic extracts were dried over Na_2SO_4 and concentrated. The crude product was purified by flash column chromatography on flash silica gel (eluted with hexanes/ethyl acetate/Et_3N 20:4:1) to afford indolenine **17** as a pale-yellow oil (1.97 g, 50% yield), the byproduct indole **19** as a pale-brown solid (1.22 g, 31% yield).

indolenine 17

R_f = 0.20 (hexanes/ethyl acetate/ Et_3N 20:4:1) [CAM].

$[\alpha]_D^{23}$ = -214.3 (c = 0.5; $CHCl_3$).

HRMS (ESI): calcd. for $C_{19}H_{23}N_2O_2$ $[M+H]^+$ 311.1760, found 311.1768.

^{1}H NMR ($CDCl_3$, 500 MHz, δ, ppm): 7.49 (1H, d, *J* = 7.7 Hz); 7.35 (1H, d, *J* = 7.5 Hz); 7.26 (1H, dd, *J* = 7.7, 7.6, Hz); 7.19 (1H, dd, *J* = 7.6, 7.5 Hz); 3.25 – 3.20 (2H, m); 3.02 (1H, br. s); 2.99 (3H, s); 2.99 (1H, m); 2.84 (1H, ddd, *J* = 15.0, 10.7, 4.6 Hz); 2.76 (1H, td, *J* = 12.5, 4.4 Hz); 2.73 (1H, m); 2.34 (1H, td, *J* = 11.6, 2.6 Hz); 2.29 (1H, td, *J* =12.5, 3.2 Hz); 2.20 (1H, m); 1.89 (1H, m); 1.80 (1H, dd, *J* = 12.4, 5.0 Hz); 1.70 (1H, dd, *J* = 13.5, 3.7 Hz); 1.58 (1H, m); 1.45 (1H, td, *J* = 13.6, 4.8 Hz).

^{13}**C NMR** ($CDCl_3$, 125 MHz, δ, ppm): 174.7; 154.3; 147.1 (2C) ; 127.3; 125.3; 120.8; 119.8; 74.3; 61.4; 54.3; 51.4; 50.9; 46.0; 34.5; 32.7; 25.9; 23.5; 21.3.

indole 19

$\mathbf{R_f}$ **=** 0.15 (hexanes/ethyl acetate/ Et_3N 20:4:1) [CAM].

$[\alpha]_D^{23}$= +164.6 (c = 0.5; $CHCl_3$).

HRMS (ESI): calcd. for $C_{19}H_{23}N_2O_2$ $[M+H]^+$ 311.1760, found 311.1749.

^{1}**H NMR** ($CDCl_3$, 500 MHz, δ, ppm): 7.74 (1H, br.s); 7.48 (1H, d, *J* = 7.2 Hz); 7.19 (1H, d, *J* = 7.4 Hz); 7.10 – 7.00 (2H, m); 3.68 (1H, br. m); 3.55 (3H, s) 3.20 – 3.00 (4H, m); 2.67 (1H, d, *J* = 6.0 Hz); 2.30 – 2.15 (2H, m); 2.10 – 1.95 (3H, m); 1.75 (1H, m); 1.72 (1H, m); 1.64 (1H, m).

^{13}**C NMR** ($CDCl_3$, 125 MHz, δ, ppm): 176.8; 136.6; 136.5; 127.4; 120.7; 118.8; 118.0; 110.2; 105.7; 67.3; 54.8; 53.0; 51.7; 46.4; 34.7; 33.0; 28.6; 23.5; 21.9.

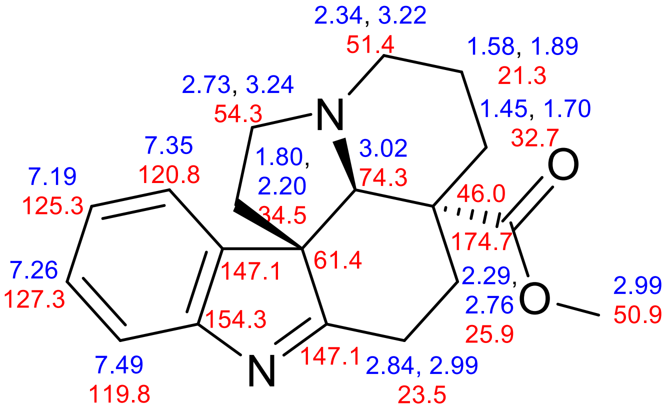
2.34, 3.22
51.4
1.58, 1.89
21.3
2.73, 3.24
54.3
N
1.45, 1.70
32.7
O
7.35
120.8
1.80,
2.20
34.5
3.02
74.3
7.19
125.3
46.0
174.7
147.1
61.4
7.26
127.3
2.29,
2.76
25.9
O
2.99
50.9
154.3
7.49
119.8
N
147.1
2.84, 2.99
23.5

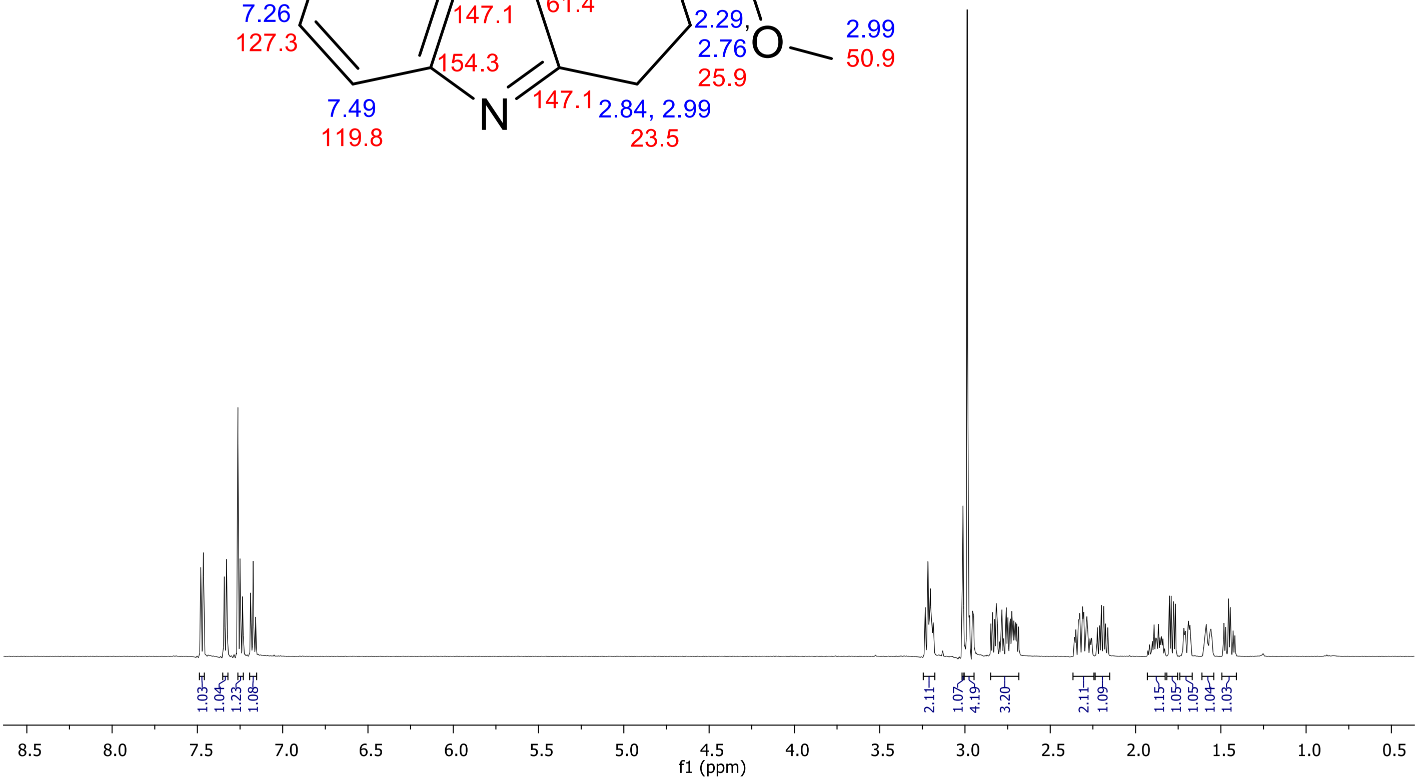
1.03
1.04
1.23
1.08
2.11
1.07
4.19
3.20
2.11
1.09
1.15
1.05
1.05
1.04
1.03
8.5
8.0
7.5
7.0
6.5
6.0
5.5
5.0
4.5
4.0
3.5
3.0
2.5
2.0
1.5
1.0
0.5
f1 (ppm)

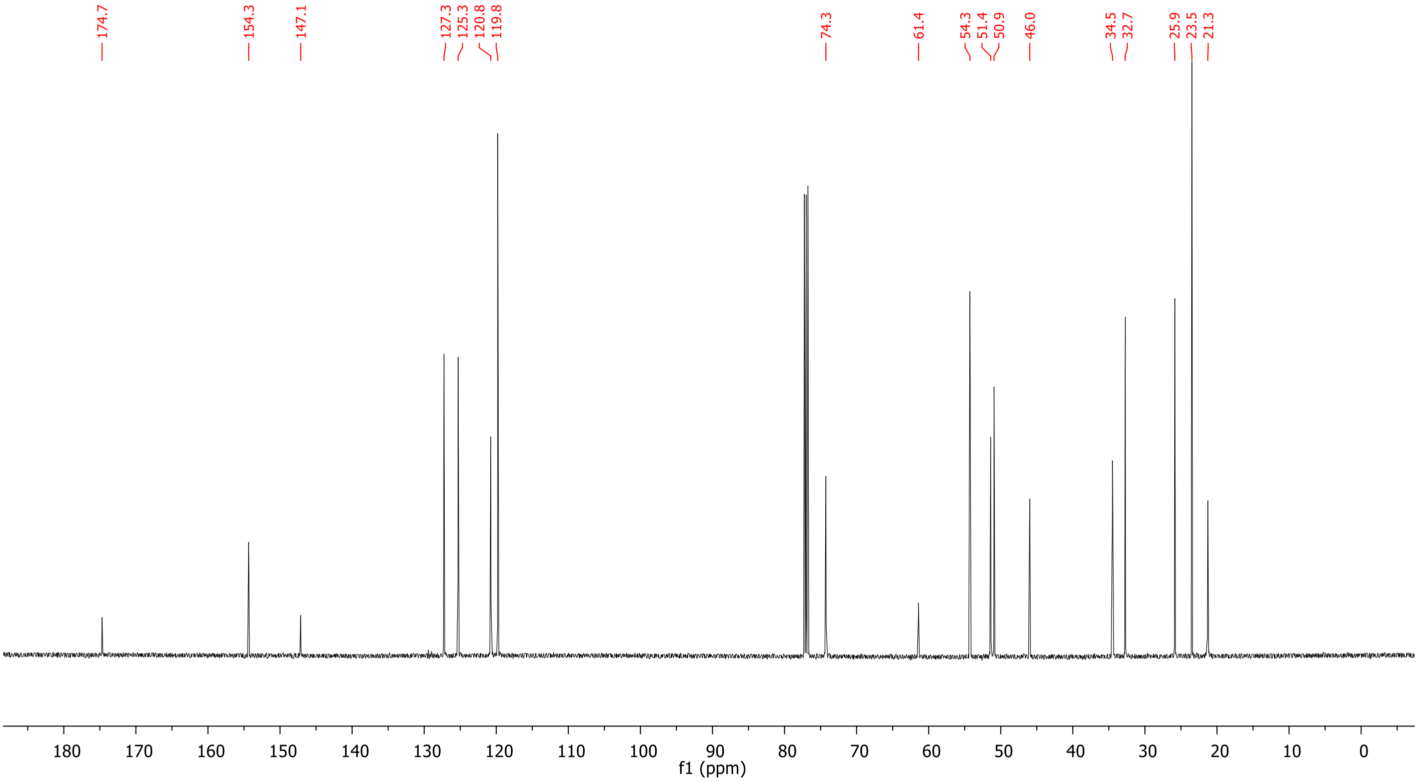
174.7
154.3
147.1
127.3
125.3
120.8
119.8
74.3
61.4
54.3
51.4
50.9
46.0
34.5
32.7
25.9
23.5
21.3
180
170
160
150
140
130
120
110
100
90
80
70
60
50
40
30
20
10
0
f1 (ppm)

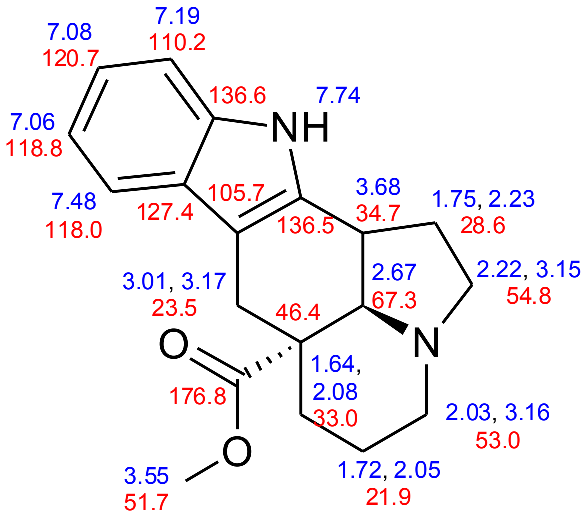

7.08
120.7
7.19
110.2
136.6
NH
7.74
7.06
118.8
7.48
118.0
127.4
105.7
136.5
3.68
34.7
1.75, 2.23
28.6
2.67
67.3
2.22, 3.15
54.8
3.01, 3.17
23.5
46.4
N
O
176.8
1.64,
2.08
33.0
2.03, 3.16
53.0
O
3.55
51.7
1.72, 2.05
21.9

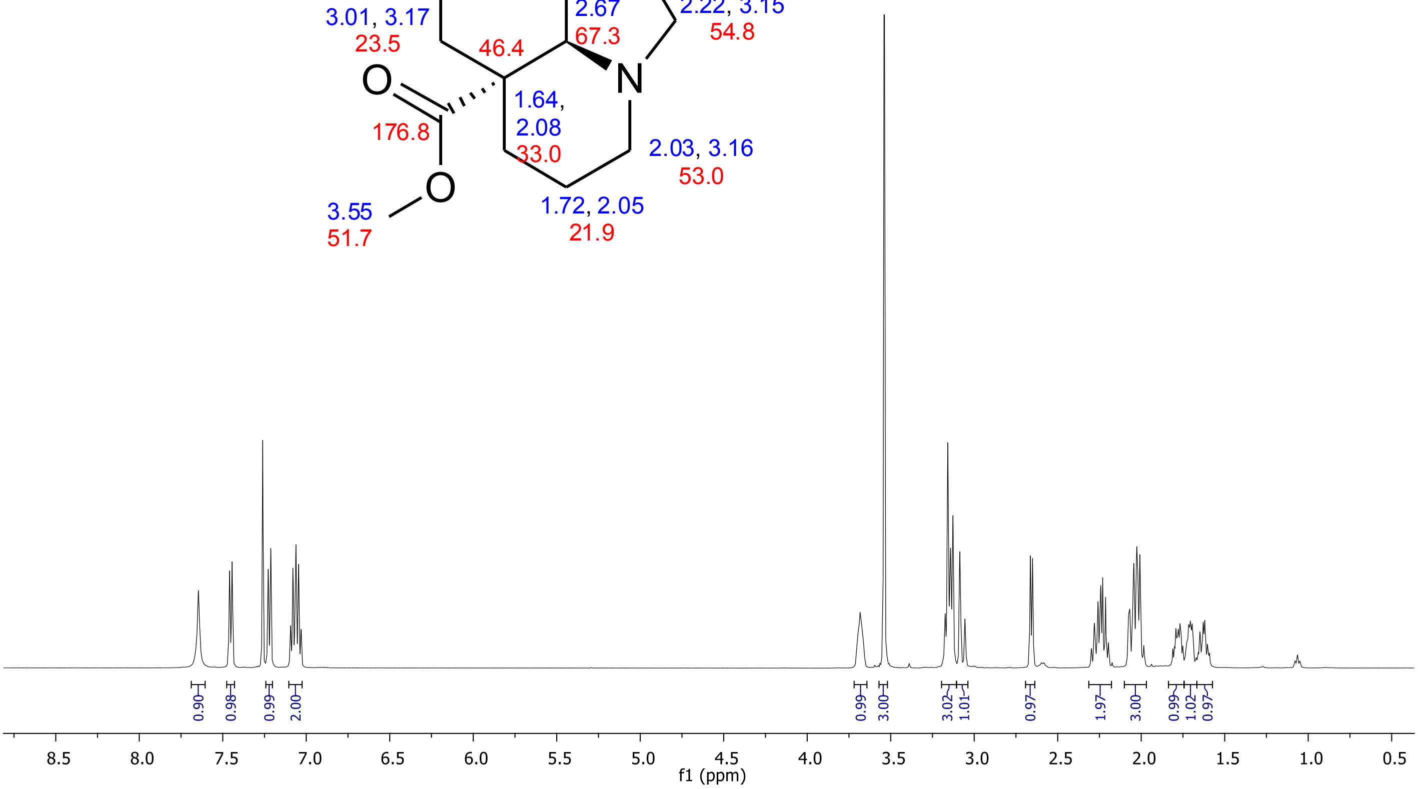

0.90
0.98
0.99
2.00
0.99
3.00
3.02
1.01
0.97
1.97
3.00
0.99
1.02
0.97
8.5 8.0 7.5 7.0 6.5 6.0 5.5 5.0 4.5 4.0 3.5 3.0 2.5 2.0 1.5 1.0 0.5
f1 (ppm)

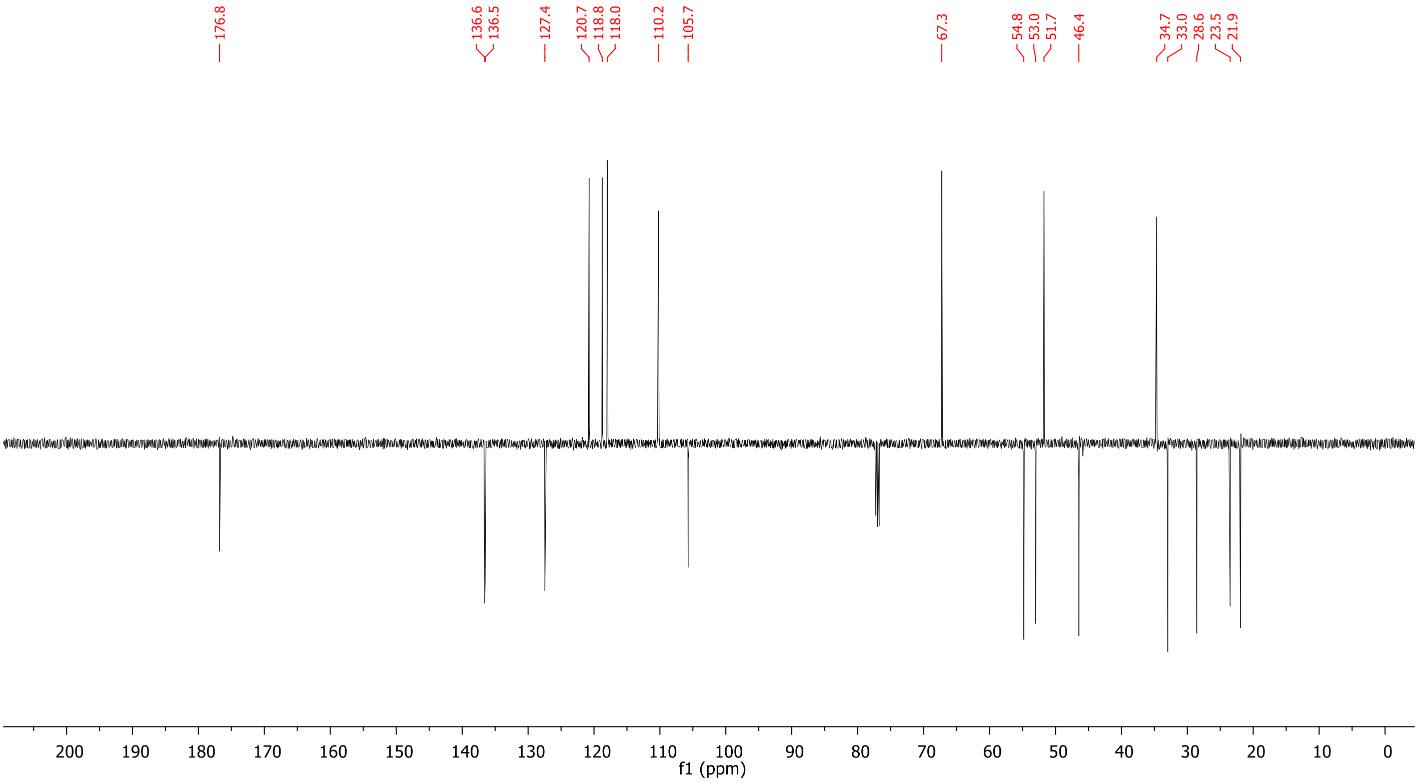

176.8
136.6
136.5
127.4
120.7
118.8
118.0
110.2
105.7
67.3
54.8
53.0
51.7
46.4
34.7
33.0
28.6
23.5
21.9
200 190 180 170 160 150 140 130 120 110 100 90 80 70 60 50 40 30 20 10 0
f1 (ppm)

Synthesis of diester 20

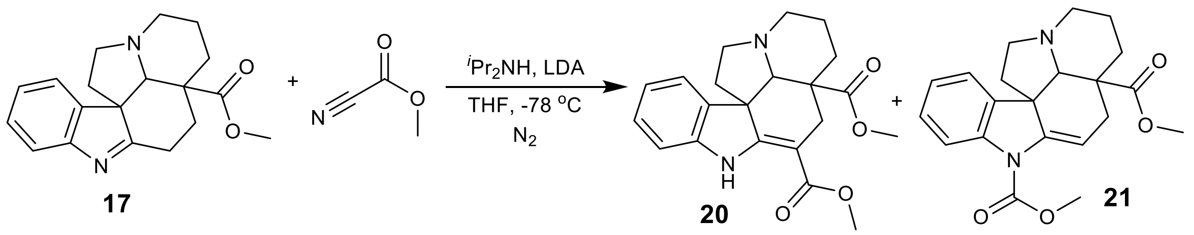

A flame dried three-necked round bottom flask equipped with a magnetic stirring bar was charged with anhydrous THF (20.0 mL) under nitrogen atmosphere and was cooled to -70 °C. Lithium diisopropylamide solution in THF (6.0 mL, 2.0 M, 2.0 eq.) was added dropwise followed by 5 minutes of stirring. To this solution was added indolenine **17** (1.86 g, 6.0 mmol, 1.00 eq.) in anhydrous THF (40.0 mL) dropwise over 20 minutes, while a change to deep yellow color was observed. The mixture was left to warm to -15 °C over 1 h and then cooled again to -70 °C. Methyl cyanoformate (1.20 ml, 15.0 mmol, 2.5 eq.) was added slowly over 5 minutes and the resulting mixture was stirred for another 20 minutes at the same temperature. After quenching with sat. aq. NH_4Cl (60 mL) the solution was left to warm to room temperature. Distilled water (20 mL) and EtOAc (60 mL) were added. After separating the phases, the watery phase was extracted with DCM (2 x 60 mL). The combined organic extracts were dried over Na_2SO_4 and concentrated. The crude product was purified by flash column chromatography on flash silica gel (eluted with hexanes/ethyl acetate 7:1–5:1) to afford the inseparable mixture of indolenine **20** and carbamate **21** (6:1 ratio, 1.95 g, Σ:88% yield) as a white foam.

$\mathbf{R_f}$ = 0.10 (hexanes/ethyl acetate 5:1) [CAM].

$[\alpha]_D^{23}$ = -403.3 (c = 1.0; $CHCl_3$).

HRMS (ESI): calcd. for $C_{21}H_{25}N_2O_4$ $[M+H]^+$ 369.1814, found 369.1823.

diester 20

^{1}H NMR ($CDCl_3$, 500 MHz, δ, ppm): 8.88 (1H, br. s); 7.27 (1H, d, J = 7.3 Hz); 7.11 (1H, t, J = 7.4 Hz); 6.89 (1H, t, J = 7.4 Hz); 6.77 (1H, d, J = 7.4 Hz); 3.76 (3H, s); 3.22 (4H, s); 3.14 (1H, dd, J = 11.6, 5.4 Hz); 2.98 – 2.90 (2H, m); 2.78 (1H, dd, J = 15.1, 1.6 Hz); 2.71 (1H, m); 2.51 (1H, td, J = 11.4, 2.4 Hz); 2.06 – 1.94 (2H, m); 1.90 – 1.75 (2H, m); 1.65 – 1.50 (2H, m).

^{13}**C NMR** ($CDCl_3$, 125 MHz, δ, ppm): 175.8; 168.6; 167.3; 142.8; 138.1; 127.0; 120.6; 120.4; 109.0; 92.4; 67.9; 56.1; 51.4; 50.9 (2C); 49.9; 48.6; 45.0; 31.7; 26.3; 22.0.

carbamate 21

^{1}**H NMR** ($CDCl_3$, 500 MHz, δ, ppm): 7.73 (1H, d, *J* = 7.5 Hz); 7.26 (1H, d, *J* = 7.5 Hz); 7.16 (1H, t, *J* = 7.5 Hz); 7.04 (1H, d, *J* = 7.5 Hz); 5.97 (1H, br. m); 3.85 (3H, s); 3.22 (4H, s); 3.12 (1H, m); 2.95 – 2.90 (2H, m); 2.50 (1H, m); 2.37 (1H, m); 2.30 (1H, m); 2.00 – 1.80 (3H, m); 1.70 (1H, m); 1.60 – 1.50 (2H, m).

^{13}**C NMR** ($CDCl_3$, 125 MHz, δ, ppm): 175.9; 168.4; 167.4; 139.6; 138.3; 126.8; 123.3; 120.2; 114.9; 108.0; 68.4; 56.2; 52.6; 51.9; 51.1; 51.0; 48.3; 44.8; 32.1; 26.5; 22.1.

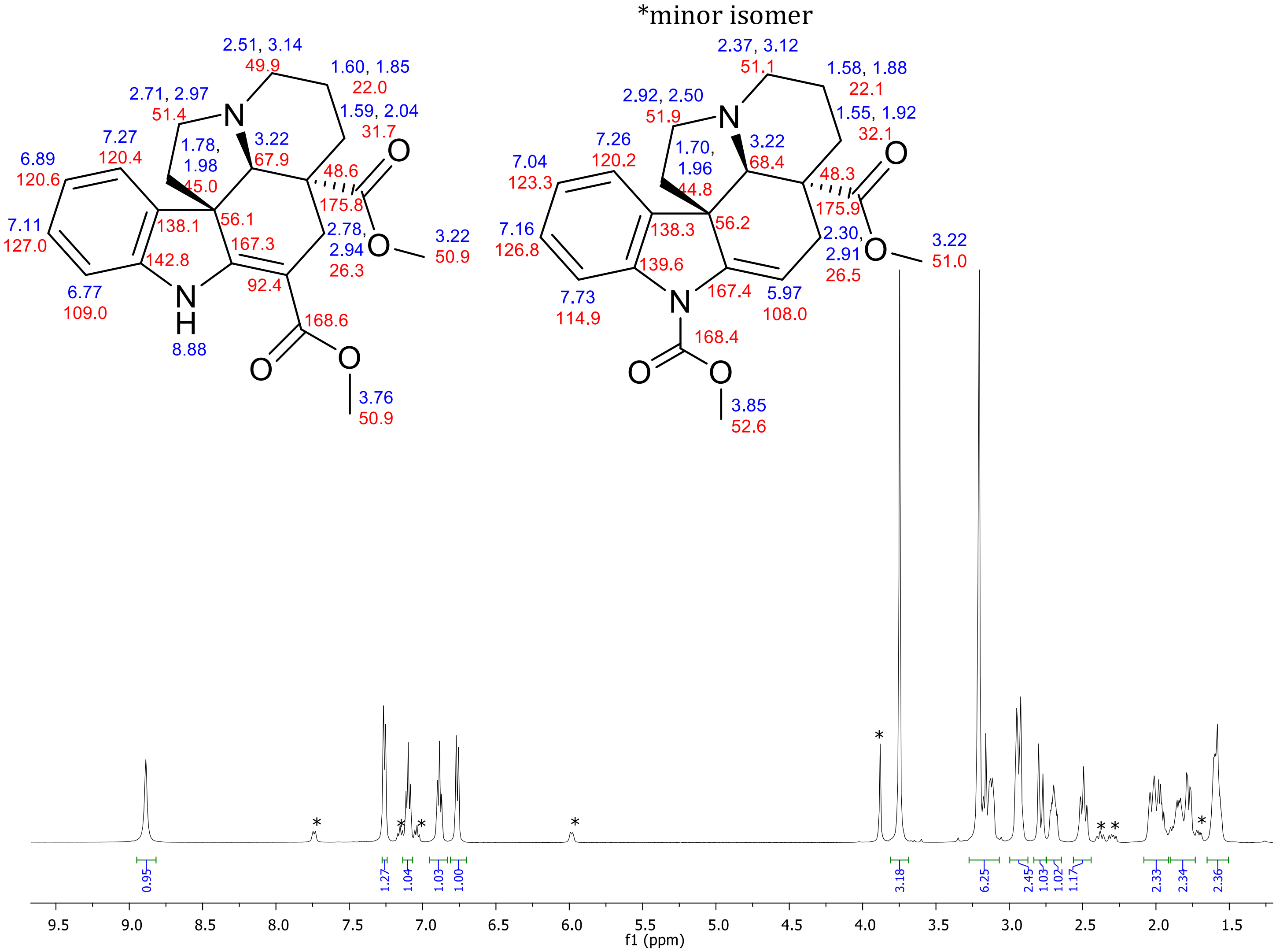
*minor isomer
2.51, 3.14
49.9
1.60, 1.85
22.0
2.71, 2.97
51.4
1.59, 2.04
31.7
7.27
120.4
1.78,
1.98
3.22
67.9
6.89
120.6
45.0
48.6
175.8
7.11
127.0
138.1
56.1
167.3
2.78,
2.94
26.3
3.22
50.9
142.8
92.4
6.77
109.0
8.88
168.6
3.76
50.9
2.37, 3.12
51.1
1.58, 1.88
22.1
2.92, 2.50
51.9
1.55, 1.92
32.1
7.26
120.2
1.70,
1.96
3.22
68.4
7.04
123.3
44.8
48.3
175.9
7.16
126.8
138.3
56.2
2.30,
2.91
26.5
3.22
51.0
139.6
7.73
114.9
167.4
5.97
108.0
168.4
3.85
52.6
0.95
1.27
1.04
1.03
1.00
3.18
6.25
2.45
1.03
1.02
1.17
2.33
2.34
2.36
9.5 9.0 8.5 8.0 7.5 7.0 6.5 6.0 5.5 5.0 4.5 4.0 3.5 3.0 2.5 2.0 1.5
f1 (ppm)

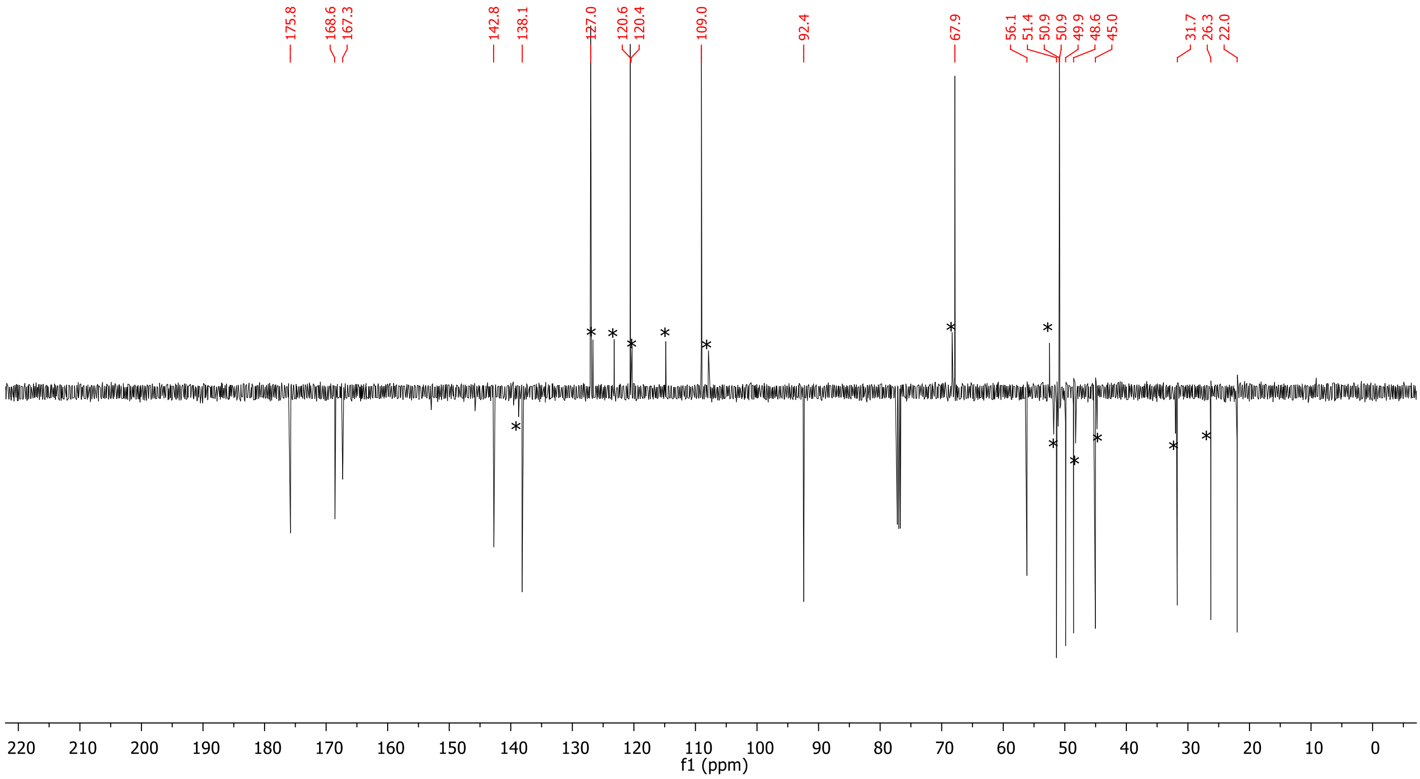
175.8
168.6
167.3
142.8
138.1
127.0
120.6
120.4
109.0
92.4
67.9
56.1
51.4
50.9
50.9
49.9
48.6
45.0
31.7
26.3
22.0
220 210 200 190 180 170 160 150 140 130 120 110 100 90 80 70 60 50 40 30 20 10 0
f1 (ppm)

Synthesis of (-)-minovincine (1)

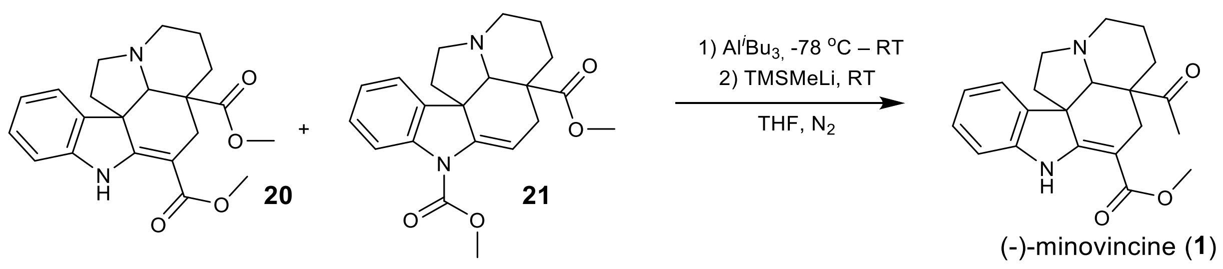

To a solution of the unseparable mixture of diester **20** and carbamate **21** (1.95 g, 5.3 mmol, 1.00 eq.) in anhydrous THF (50 mL) was added Al^{i}Bu$_3$ in toluene (5.85 mL, 1.0 M, 1.1 eq.) at -78 °C under nitrogen atmosphere. The reaction mixture was allowed to warm to room temperature and stirred for 1 h, while a change to bright yellow colour was experienced. (Trimethylsilyl)methyllithium in pentane (31.9 mL, 1.0 M, 6.1 eq.) was added with a syringe pump over 3 h and then the reaction mixture was stirred for an additional 30 min. MeOH (25 mL) was added dropwise, consequently the brown reaction mixture turned yellow. After stirring for 20 minutes, sat. aq. Rochelle salt solution (50 mL) was added and vigorous stirring was maintained for 14 h. The mixture was then treated with sat. aq. $NaHCO_3$ (150 mL) and extracted with DCM (3 x 150 mL). The combined organic extracts were dried over Na_2SO_4 and concentrated. The crude product was purified by flash column chromatography on flash silica gel (eluted with hexanes/ethyl acetate/Et_3N 21:3:1) to afford (-)-minovincine (**1**) as white crystals (1.10 g, 68% yield [52% yield over two steps]) and recovered diester **20** (334 mg, 17% yield).

All analytical data were in accordance with data reported in the literature.[3]

R$_f$ = 0.35 (hexanes/ethyl acetate/ Et_3N 10:2:1) [CAM].

$[\alpha]_D^{23}$ = -496.9 (c = 1.2; $CHCl_3$).

HRMS (ESI): calcd. for $C_{21}H_{25}N_2O_3$ $[M+H]^+$ 353.1865, found 353.1880.

^{1}H NMR ($CDCl_3$, 500 MHz, δ, ppm): 8.78 (1H, br. s); 7.33 (1H, d, *J* = 9.0 Hz); 7.12 (1H, t, *J* = 8.2 Hz); 6.93 (1H, dd, *J* = 9.0, 8.2 Hz); 6.77 (1H, d, *J* = 8.2 Hz); 3.77 (3H, s); 3.31 (1H, s); 3.14 (1H, m); 3.03 (1H, d, *J* = 14.2 Hz); 2.98 (1H, m); 2.81 (1H, d, *J* = 14.2 Hz); 2.76 (1H,

[3] B. N. Laforteza, M. Pickworth, D. W. C. MacMillan, *Angew. Chem.* **2013**, *125*, 11479–11482; *Angew. Chem. Int. Ed.* **2013**, *52*, 11269–11272.

m); 2.51 (1H, m); 1.98 (1H, m); 1.90 (1H, m); 1.88 (3H, s); 1.78 (1H, m); 1.64 (1H, d, *J* = 13.0 Hz); 1.42 (1H, t, *J* = 12.2 Hz).

^{13}**C NMR** ($CDCl_3$, 125 MHz, δ, ppm): 212.0; 168.3, 168.2; 142.5; 138.2; 127.2; 121.0; 120.7; 109.4; 91.5; 67.7; 56.2; 53.9; 51.5; 51.1; 49.8; 45.3; 31.1; 25.9; 25.1; 22.4.

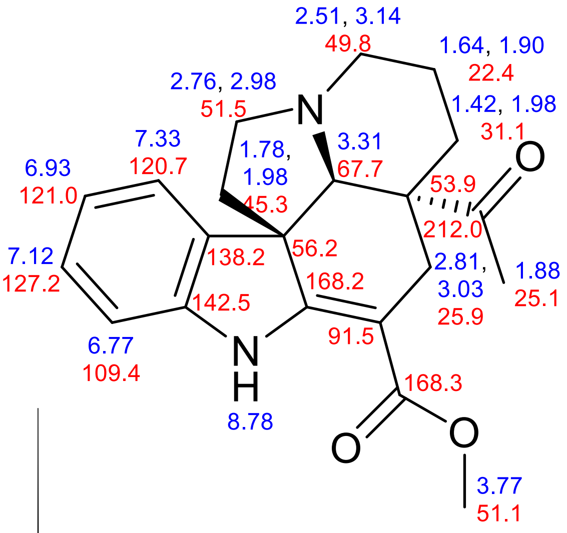
2.51, 3.14
49.8
1.64, 1.90
22.4
2.76, 2.98
51.5
1.42, 1.98
31.1
7.33
120.7
1.78,
1.98
45.3
3.31
67.7
6.93
121.0
53.9
212.0
7.12
127.2
138.2
56.2
168.2
2.81,
3.03
25.9
1.88
25.1
142.5
6.77
109.4
91.5
168.3
8.78
3.77
51.1

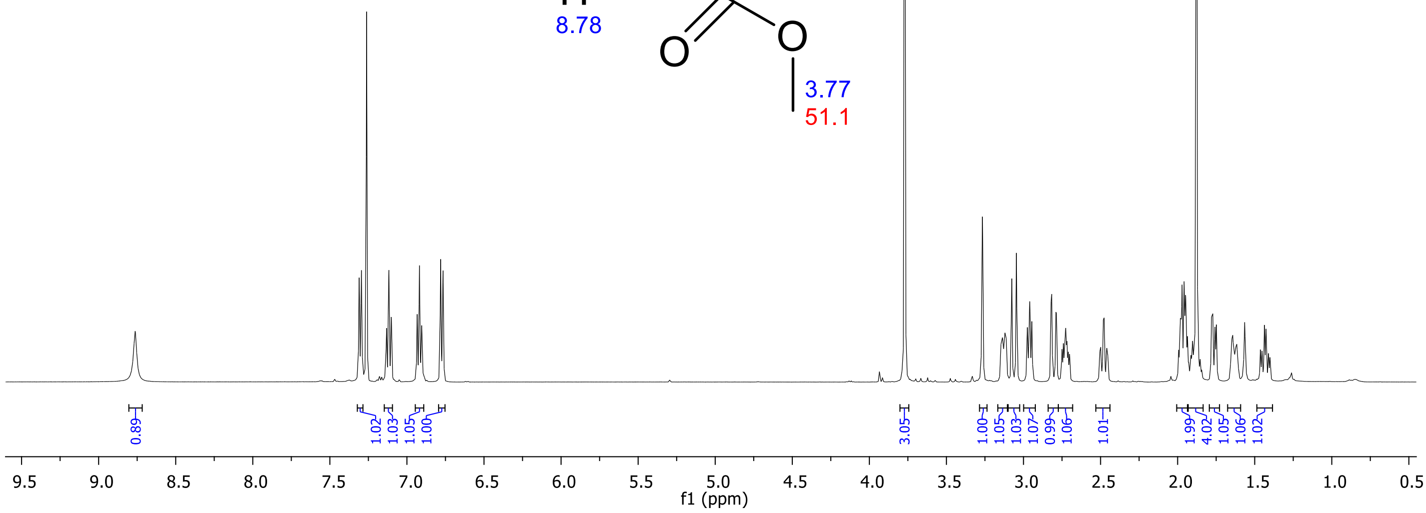
0.89
1.02
1.03
1.05
1.00
3.05
1.00
1.05
1.03
1.07
0.99
1.06
1.01
1.99
4.02
1.05
1.06
1.02
9.5 9.0 8.5 8.0 7.5 7.0 6.5 6.0 5.5 5.0 4.5 4.0 3.5 3.0 2.5 2.0 1.5 1.0 0.5
f1 (ppm)

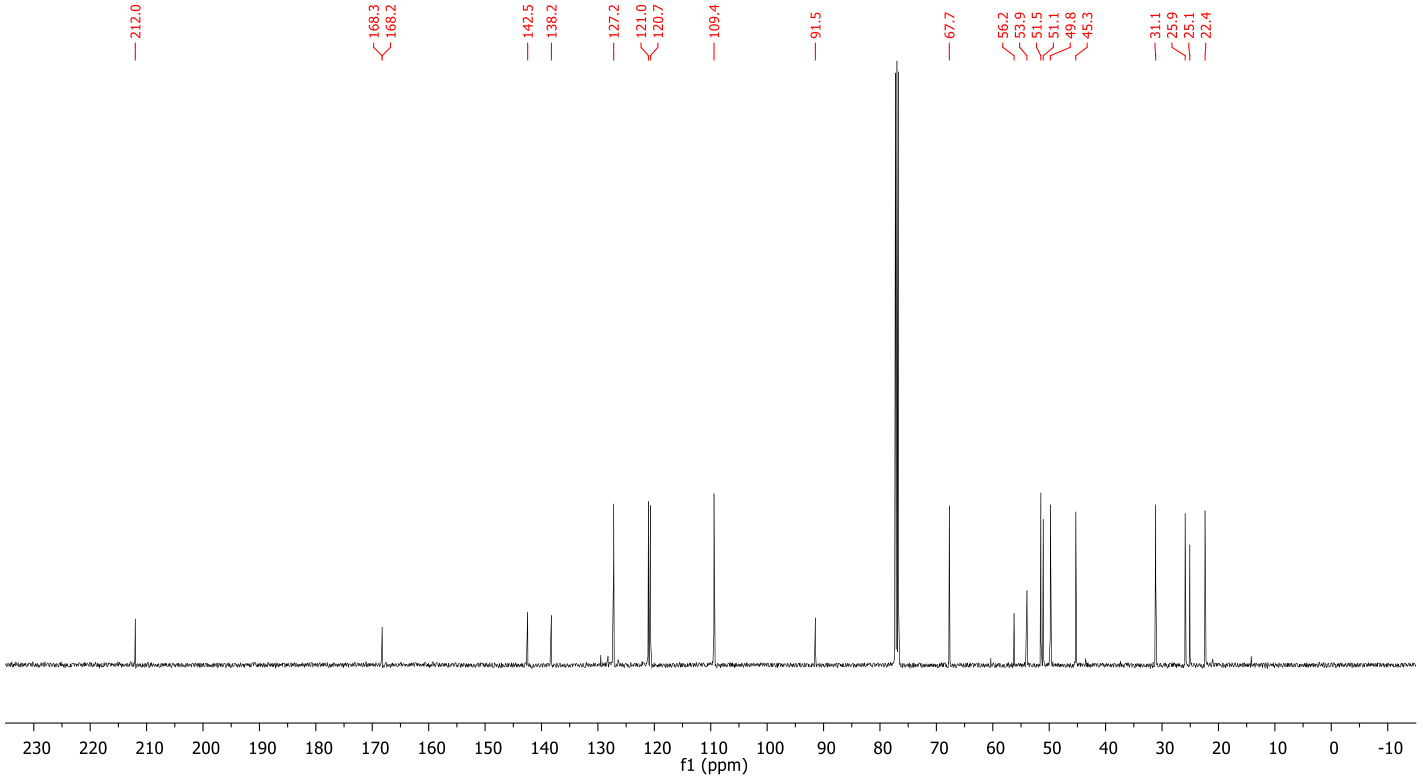
212.0
168.3
168.2
142.5
138.2
127.2
121.0
120.7
109.4
91.5
67.7
56.2
53.9
51.5
51.1
49.8
45.3
31.1
25.9
25.1
22.4
230 220 210 200 190 180 170 160 150 140 130 120 110 100 90 80 70 60 50 40 30 20 10 0 -10
f1 (ppm)

Synthesis of diketone 23

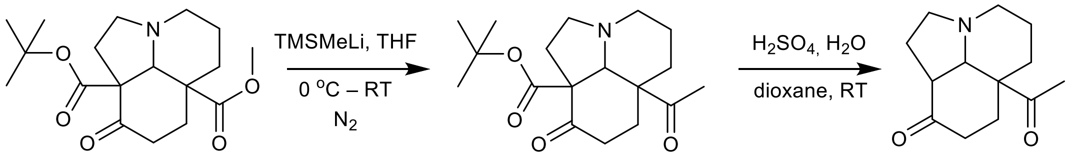

A flame dried three-necked round bottom flask equipped with a magnetic stirring bar was charged with anhydrous THF (40.0 ml) under a nitrogen atmosphere. Ketone **8** (2.02 g, 6.02 mmol, 1.00 eq.) was added and the solution was cooled to -5 °C. (Trimethylsilyl)methyllithium in pentane (25.0 mL, 1.0 M, 4.15 eq.) was added via syringe over 20 minutes while keeping the internal temperature below 0 °C. The brown solution was kept stirring at 0 °C. The reaction was monitored by GC-MS analysis. A small sample was withdrawn from the solution in every 40 minutes, quenched with sat. aq. Na_2CO_3 and extracted with EtOAc. If no starting material was observed (typically after less than 2 h), then MeOH (6.0 mL) was added slowly via syringe to the solution, while maintaining the temperature below 5 °C. (NOTE: If starting material is still observed in the sample, further (trimethylsilyl)methyllithium (6.0 mL, 1.0 M, 1.00 eq.) addition may also help to reach full conversion before quenching with MeOH.) Bright yellow colour, salt formation upon MeOH addition, which dissolved by further MeOH was observed during quenching. The cooling bath was then removed and the solution was left to warm to room temperature and stirred for 1 h. The mixture was then treated with sat. aq. Na_2CO_3 (50 mL) and extracted with EtOAc (3 x 40 mL). The combined organic extracts were dried over Na_2SO_4 and concentrated. The desired product was obtained as a crude yellow oil (1.96 g) and used in the next step without further purification.

The crude oil (1.96 g) was dissolved in dioxane (12.0 mL) and aq. H_2SO_4 (12.0 mL, 50 *V*/*V*%) was added slowly while stirring vigorously. The solution was stirred for further 14 h at room temperature. The solution was then treated with sat. aq. Na_2CO_3 until reaching pH = 10 (NOTE: harsh gas evolution was observed) and extracted with DCM (3 x 40 mL). The combined organic extracts were dried over Na_2SO_4 and concentrated. A brown crude oil (1.20 g) was obtained which was purified by flash column chromatography on flash silica gel

(eluted with hexanes/ethyl acetate 2:1) to afford diketone **23** as a yellow oil (1.09 g, 80% yield for the 2 steps).

R_f = 0.20 (hexanes/ethyl acetate 2:1) [CAM].

$[\alpha]_D^{23}$ = +45.9 (c = 1.0; $CHCl_3$).

HRMS (ESI): calcd. for $C_{13}H_{20}NO_2$ $[M+H]^+$ 222.1494, found 222.1491.

^{1}H NMR ($CDCl_3$, 500 MHz, δ, ppm): 3.00 (2H, m); 2.91 (1H, ddd, *J* = 9.3, 5.3, 2.0 Hz); 2.53 (2H, m); 2.40 (1H, m); 2.36 (1H, dt, *J* = 14.2, 3.3 Hz); 2.24 (3H, s); 2.09 (1H, td, *J* = 14.2, 5.3 Hz); 2.00 – 1.80 (5H, m); 1.69 (1H, m); 1.62 (1H, m); 1.40 (1H, m).

^{13}CNMR ($CDCl_3$, 125 MHz, δ, ppm): 211.9; 209.7; 67.4; 52.4; 52.3; 51.0; 49.1; 38.1; 31.5; 26.5; 24.4; 21.2 (2C).

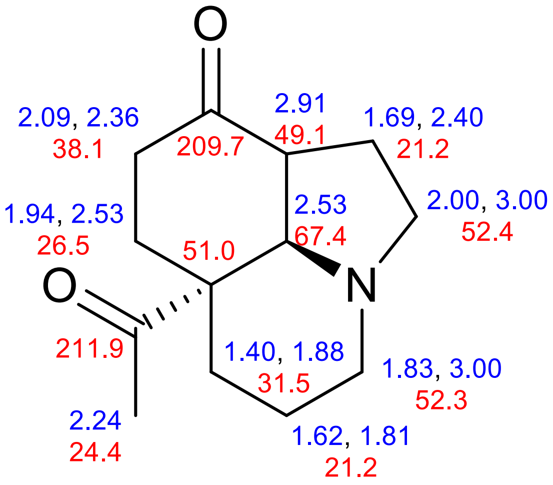
O
2.09, 2.36
38.1
209.7
2.91
49.1
1.69, 2.40
21.2
1.94, 2.53
26.5
2.53
67.4
2.00, 3.00
52.4
51.0
O
N
211.9
1.40, 1.88
31.5
1.83, 3.00
52.3
2.24
24.4
1.62, 1.81
21.2

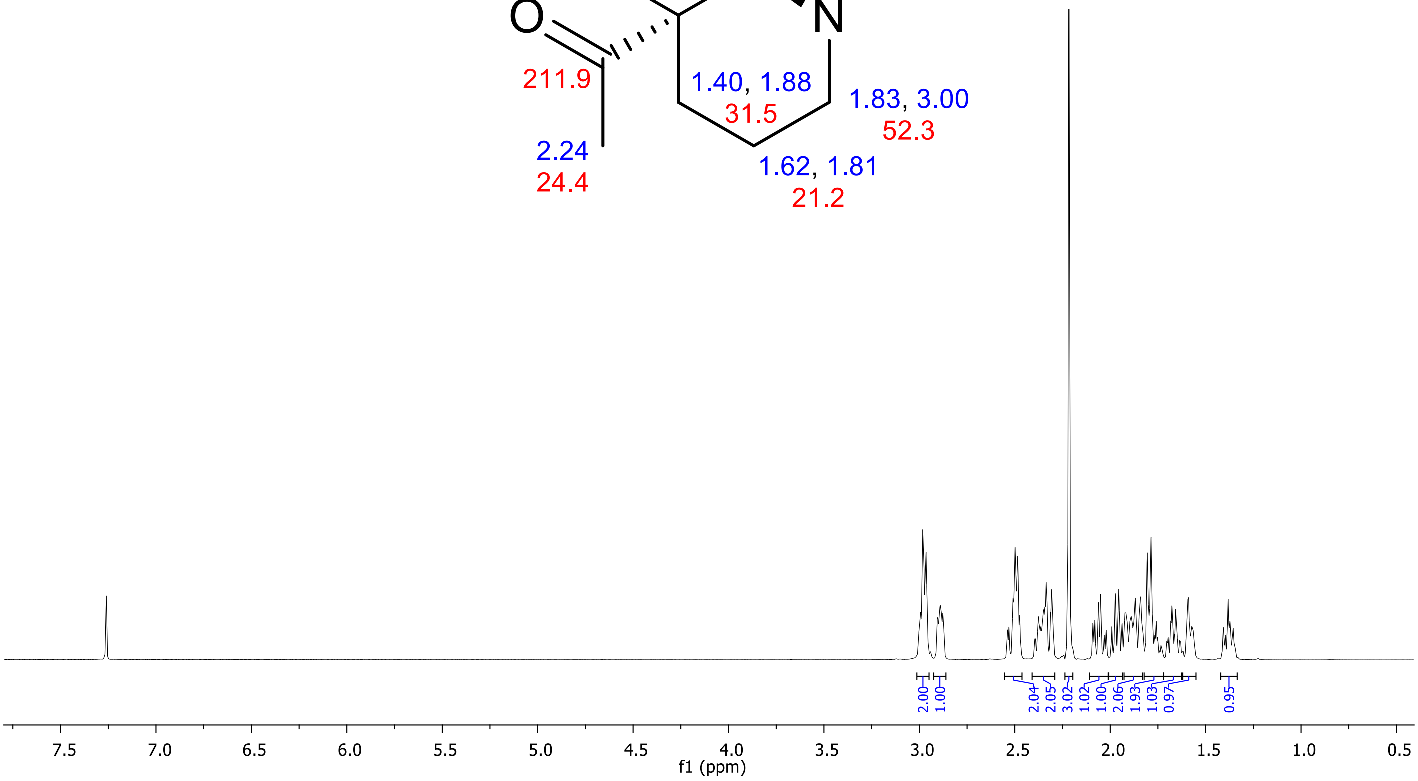
2.00
1.00
2.04
2.05
3.02
1.02
1.00
2.06
1.93
1.03
0.97
0.95
7.5 7.0 6.5 6.0 5.5 5.0 4.5 4.0 3.5 3.0 2.5 2.0 1.5 1.0 0.5
f1 (ppm)

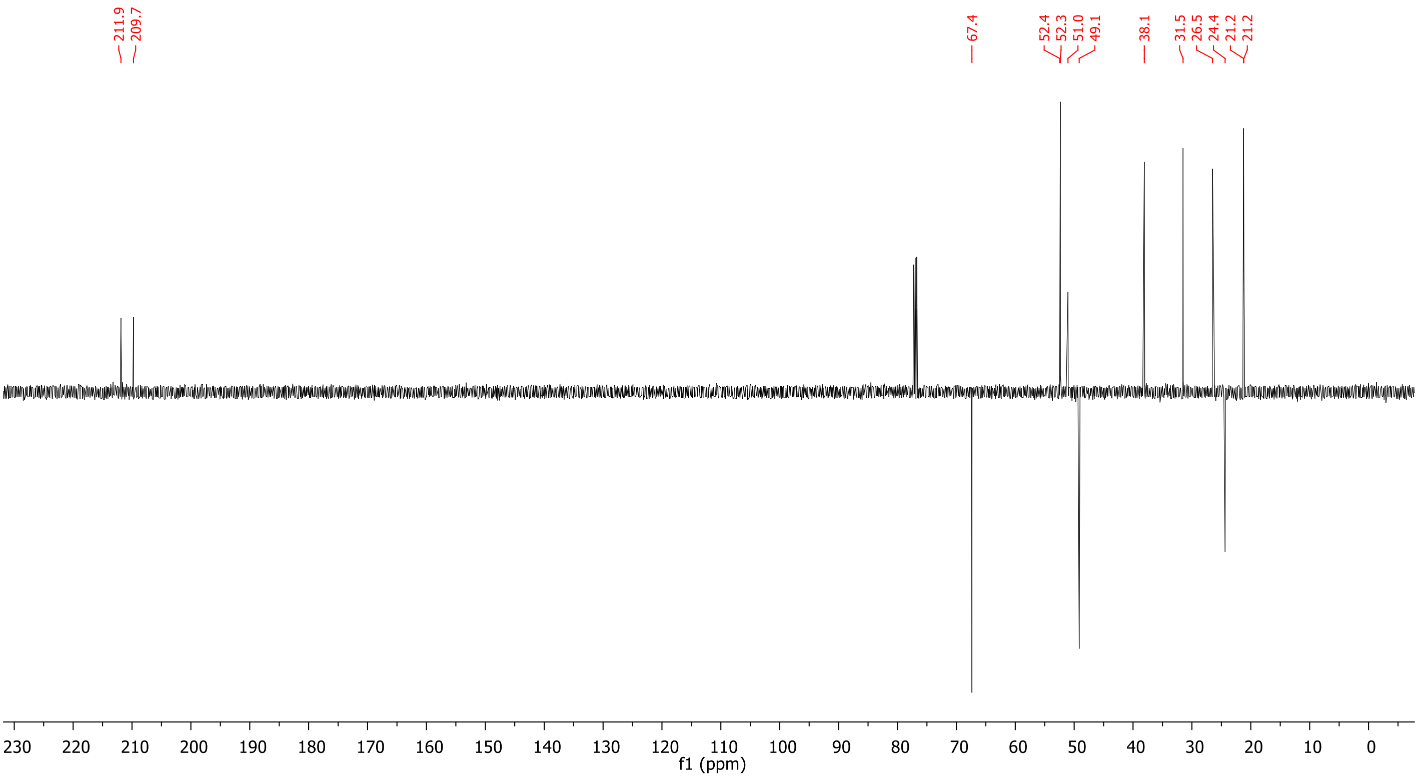
211.9
209.7
67.4
52.4
52.3
51.0
49.1
38.1
31.5
26.5
24.4
21.2
21.2
230 220 210 200 190 180 170 160 150 140 130 120 110 100 90 80 70 60 50 40 30 20 10 0
f1 (ppm)

Synthesis of oxo-aspidofractinine 25

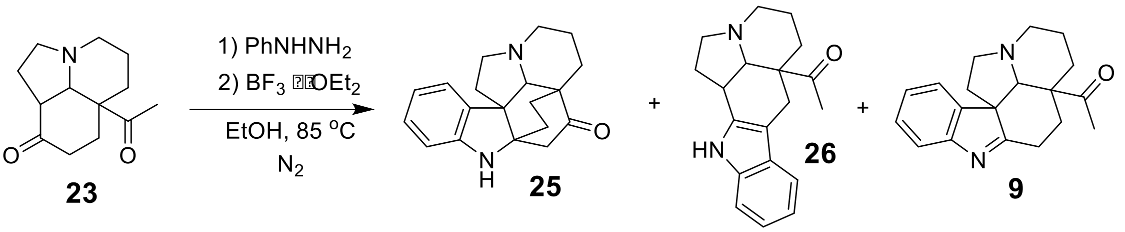

To a solution of **23** (350 mg, 1.58 mmol, 1.00 eq.) in EtOH (7.0 mL) in a sealed tube was added $PhNHNH_2$ (163 µl, 1.66 mmol, 1.05 eq.). The reaction mixture was placed in a preheated oil bath at 85 °C and stirred for 2 h under nitrogen atmosphere. The reaction mixture was cooled to room temperature and $BF_3{\cdot}OEt_2$-EtOH solution (21 mL, 1:9 V/V) was added in one portion. The resulting mixture was placed in a preheated oil bath at 85 ^{o}C and stirred for further 16 h. The resulting brown solution was cooled to room temperature, treated with sat. aq. Na_2CO_3 (40 mL) and extracted with DCM (3 x 40 mL). The combined organic extracts were dried over Na_2SO_4 and concentrated. The crude product was purified by flash column chromatography on flash silica gel (eluted with hexanes/ethyl acetate/Et_3N 12:4:1) to afford indoline **25** as a pale-yellow solid (255 mg, 55% yield), the byproduct indole **26** as a pale-brown oil (130 mg, 28% yield) and the some indolenine **9** as a yellow oil (37 mg, 8% yield).

indoline 25

All analytical data were in accordance with data reported in the literature.[4]

R_f = 0.50 (hexanes/ethyl acetate/ Et_3N 8:8:1) [CAM].

$[\boldsymbol{\alpha}]_{\boldsymbol{D}}^{\mathbf{23}}$ = +78.6 (c = 1.0; $CHCl_3$).

HRMS (ESI): calcd. for $C_{19}H_{23}N_2O$ $[M+H]^+$ 295.1810, found 295.1813.

^{1}H NMR ($CDCl_3$, 500 MHz, δ, ppm): 7.21 (1H, d, *J* = 7.5 Hz); 7.03 (1H, t, *J* = 8.0 Hz); 6.78 (1H, dd, *J* = 8.0, 7.5 Hz); 6.67 (1H, d, *J* = 8.0 Hz); 3.62 (1H, br.s); 3.24 (1H, m); 3.14 (1H, m); 3.13 (1H, s); 3.03 (1H, m); 2.89 (1H, td, *J* = 12.8, 2.0 Hz); 2.72 (1H, ddd, *J* = 12.9, 7.8, 5.2 Hz); 2.61 (1H, dd, *J* = 18.7, 3.1 Hz); 2.55 (1H, ddd, *J* = 13.6, 12.0, 6.3 Hz); 2.39 (1H, ddd, *J* =12.1, 11.8, 6.3 Hz); 2.00 (1H, d, *J* = 18.7 Hz); 1.82 – 1.62 (5H, m); 1.48 (1H, m); 1.25 (1H, m).

---

[4] M. Dufour, J-C. Gramain, H.-P. Husson, M.-E. Sinibaldi, Y. Troin, *Tetrahedron Lett.* **1989**, *30*, 3429–3432

^{13}**C NMR** ($CDCl_3$, 125 MHz, δ, ppm): 212.7; 148.8; 138.7; 127.4; 122.0; 120.4; 111.2; 66.9; 65.0; 56.9; 51.0; 48.3; 47.5; 46.6; 35.2; 27.0; 26.0; 23.7; 17.0.

indole 26

$\mathbf{R_f}$ = 0.60 (hexanes/ethyl acetate/ Et_3N 8:8:1) [CAM].

$[\alpha]_D^{23}$ = +118.3 (c = 0.6; $CHCl_3$).

HRMS (ESI): calcd. for $C_{19}H_{23}N_2O$ $[M+H]^+$ 295.1810, found 295.1820.

^{1}**H NMR** ($CDCl_3$, 500 MHz, δ, ppm): 7.80 (1H, br. s); 7.46 (1H, d, *J* = 7.7 Hz); 7.20 (1H, d, *J* = 7.1 Hz); 7.10 – 7.00 (2H, m); 3.59 (1H, m); 3.20 (1H, d, *J* = 15.9 Hz); 3.18 – 3.10 (2H, m); 3.00 (1H, d, *J* = 15.9 Hz); 2.64 (1H, d, *J* = 6.7 Hz); 2.27 – 2.15 (2H, m); 2.05 (1H, m); 2.04 (3H, s); 2.02 – 1.95 (2H, m); 1.80 – 1.60 (2H, m); 1.48 (1H, td, *J* = 13.1, 5.2 Hz).

^{13}**C NMR** ($CDCl_3$, 125 MHz, δ, ppm): 212.9; 137.4; 136.5; 127.3; 121.0; 119.0; 117.6; 110.4; 105.4; 67.1; 54.9; 53.0; 52.2; 34.2; 32.2; 28.9; 25.3; 23.3; 22.1

indolenine 9

All analytical data were in accordance with data reported in the literature.[4]

$\mathbf{R_f}$ = 0.40 (hexanes/ethyl acetate/ Et_3N 8:8:1) [CAM].

$[\alpha]_D^{23}$ = -211.4 (c = 0.5; $CHCl_3$).

HRMS (ESI): calcd. for $C_{19}H_{23}N_2O$ $[M+H]^+$ 295.1810, found 295.1798.

^{1}**H NMR** ($CDCl_3$, 500 MHz, δ, ppm): 7.47 (1H, d, *J* = 7.6 Hz); 7.37 (1H, d, *J* = 7.4 Hz); 7.28 (1H, td, *J* = 7.5, 1.2 Hz); 7.19 (1H, td, *J* = 7.5, 1.0 Hz); 3.27 – 3.17 (2H, m); 3.06 (1H, s); 3.00 (1H, m); 2.90 – 2.78 (2H, m); 2.74 (1H, ddd, *J* = 11.4, 8.7, 5.2 Hz); 2.33 (1H, td, *J* = 12.0, 3.2 Hz); 2.28 – 2.13 (2H, m); 1.90 (1H, m); 1.76 (1H, dd, *J* = 12.4, 5.1 Hz); 1.69 (3H, s); 1.66 – 1.52 (2H, m); 1.29 (1H, td, *J* = 13.3, 5.7 Hz).

^{13}**C NMR** ($CDCl_3$, 125 MHz, δ, ppm): 209.4; 188.5; 154.2; 147.1; 127.7; 125.5; 120.8; 120.3; 73.9; 61.4; 54.2; 52.1; 51.3; 35.0; 32.2; 25.6; 25.0; 23.6; 21.4.

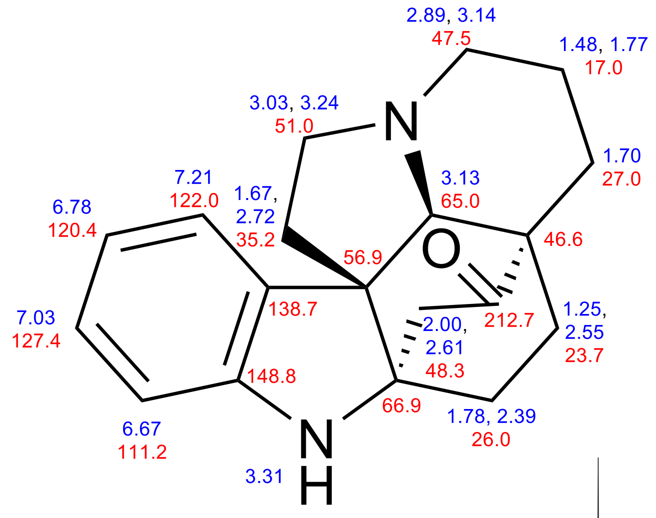
2.89, 3.14
47.5
1.48, 1.77
17.0
3.03, 3.24
51.0
N
1.70
27.0
7.21
122.0
1.67,
2.72
35.2
3.13
65.0
6.78
120.4
46.6
56.9
O
138.7
7.03
127.4
2.00,
2.61
48.3
212.7
1.25,
2.55
23.7
148.8
66.9
1.78, 2.39
26.0
6.67
111.2
NH
3.31

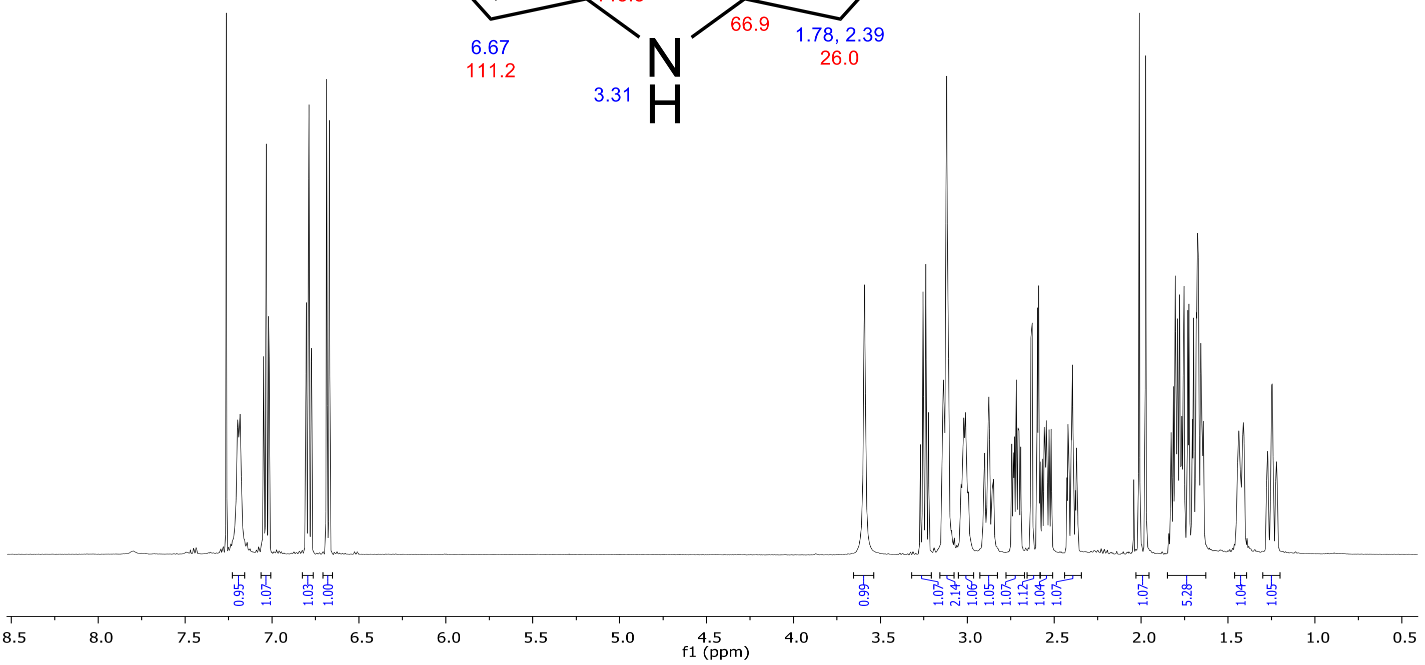
0.95
1.07
1.03
1.00
0.99
1.07
2.14
1.06
1.05
1.07
1.12
1.04
1.07
1.07
5.28
1.04
1.05
f1 (ppm)

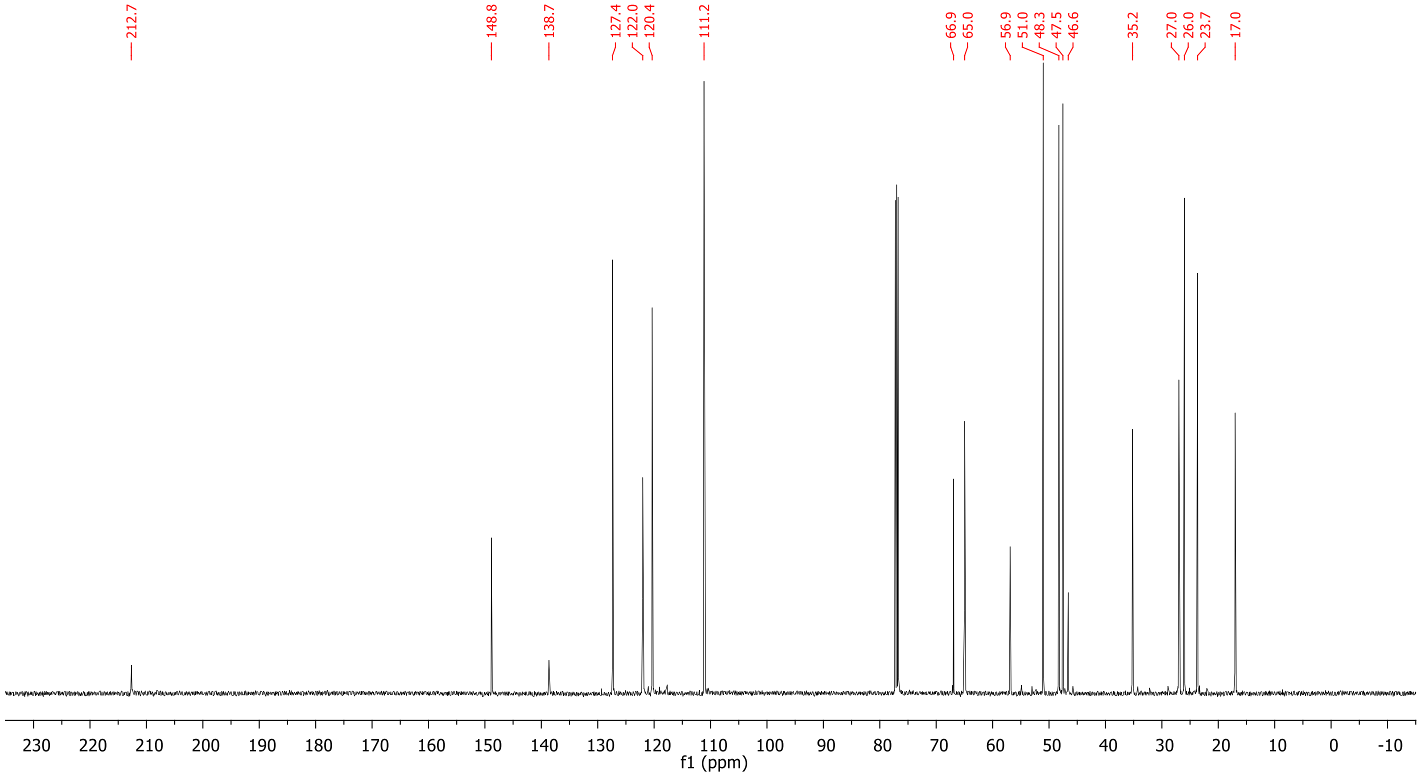
212.7
148.8
138.7
127.4
122.0
120.4
111.2
66.9
65.0
56.9
51.0
48.3
47.5
46.6
35.2
27.0
26.0
23.7
17.0
f1 (ppm)

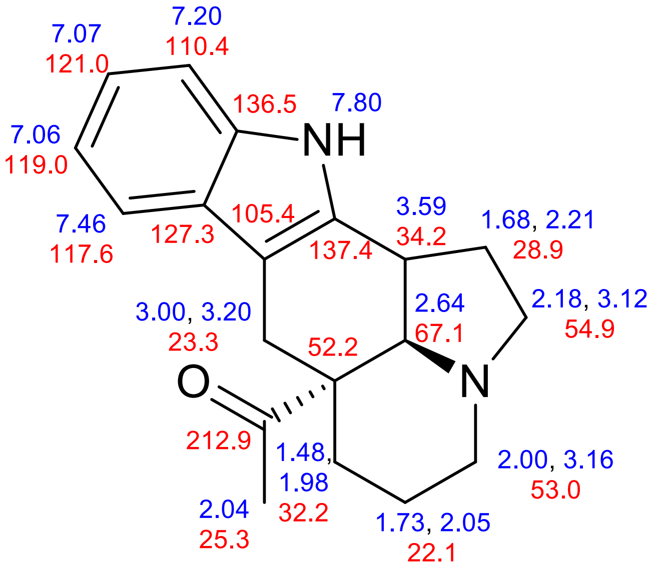
7.07
121.0
7.20
110.4
136.5
7.80
NH
7.06
119.0
7.46
117.6
127.3
105.4
137.4
3.59
34.2
1.68, 2.21
28.9
3.00, 3.20
23.3
2.64
67.1
2.18, 3.12
54.9
52.2
O
N
212.9
1.48,
1.98
32.2
2.00, 3.16
53.0
2.04
25.3
1.73, 2.05
22.1

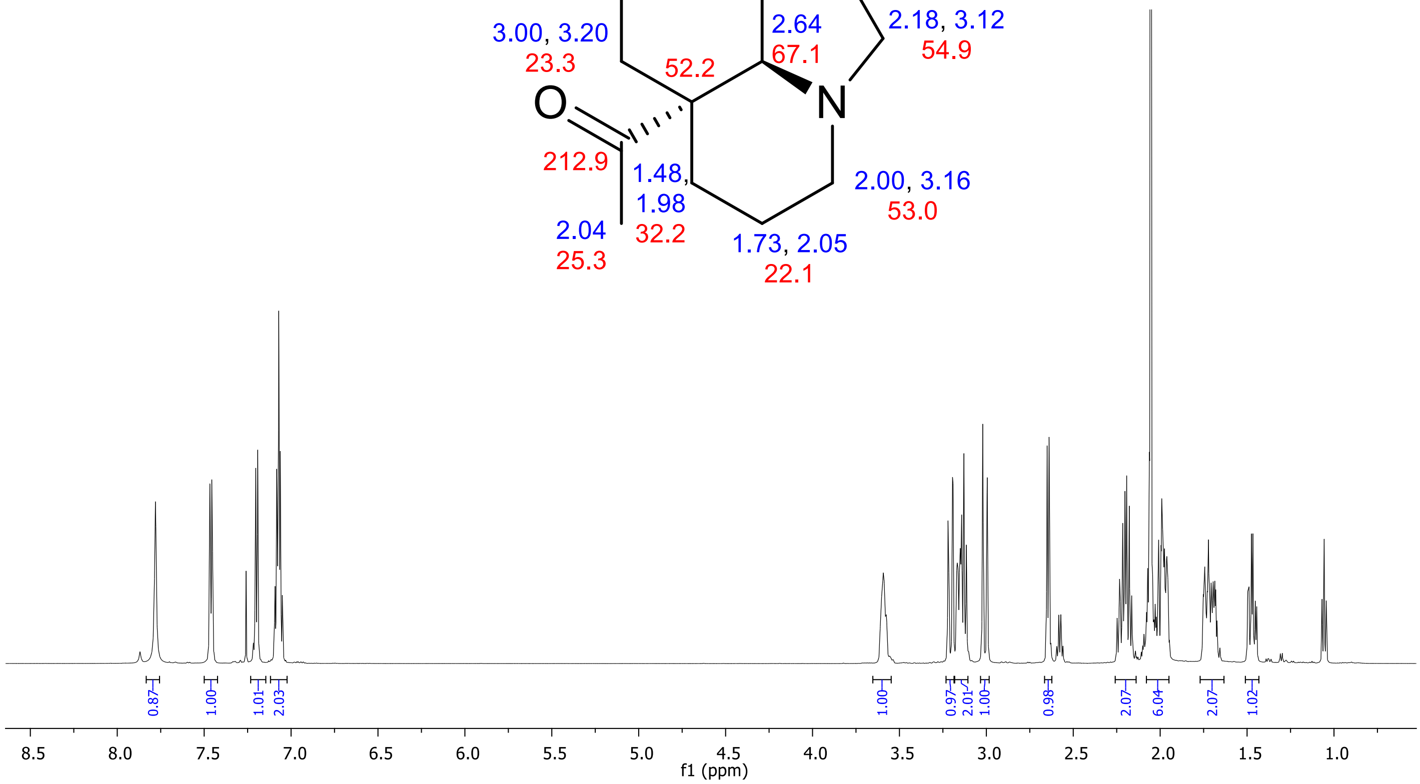
0.87
1.00
1.01
2.03
1.00
0.97
2.01
1.00
0.98
2.07
6.04
2.07
1.02
8.5
8.0
7.5
7.0
6.5
6.0
5.5
5.0
4.5
4.0
3.5
3.0
2.5
2.0
1.5
1.0
f1 (ppm)

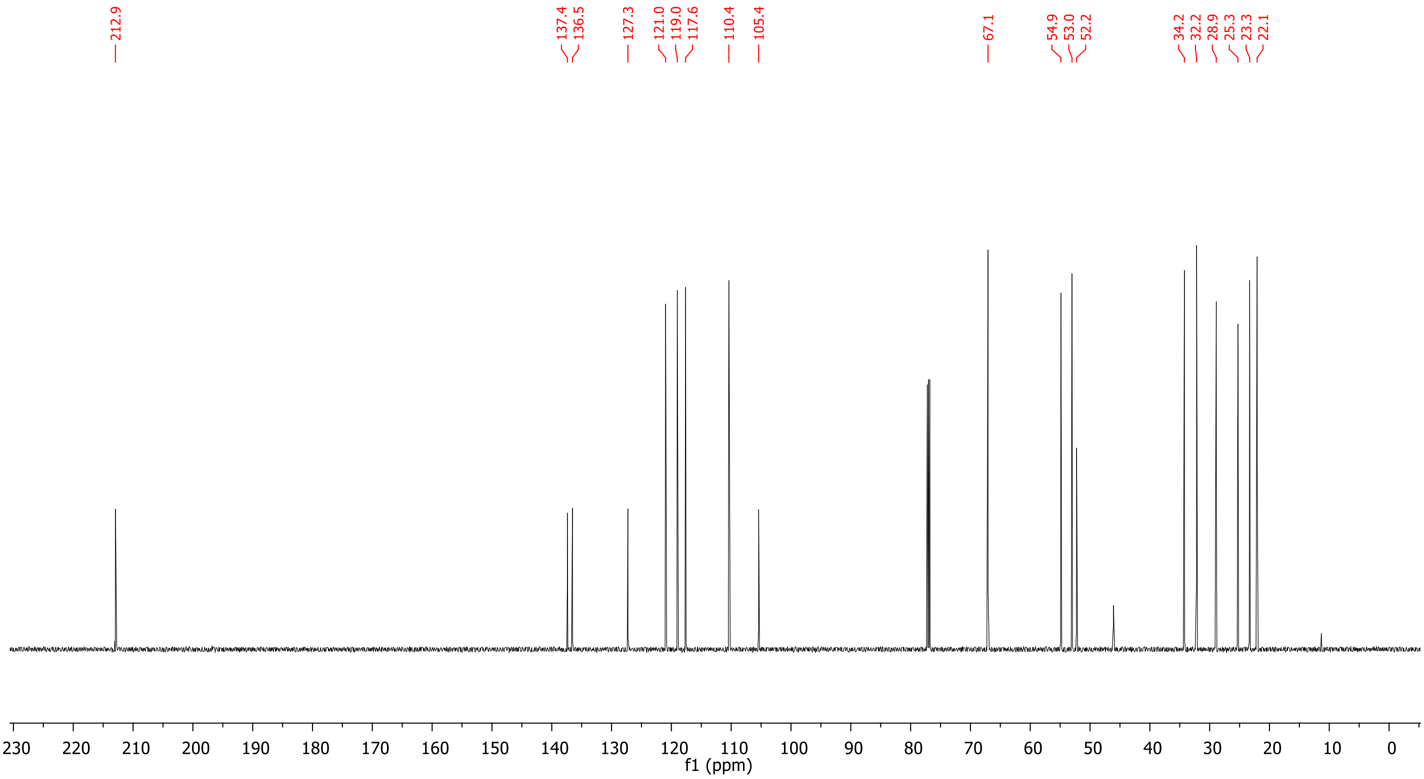
212.9
137.4
136.5
127.3
121.0
119.0
117.6
110.4
105.4
67.1
54.9
53.0
52.2
34.2
32.2
28.9
25.3
23.3
22.1
230
220
210
200
190
180
170
160
150
140
130
120
110
100
90
80
70
60
50
40
30
20
10
0
f1 (ppm)

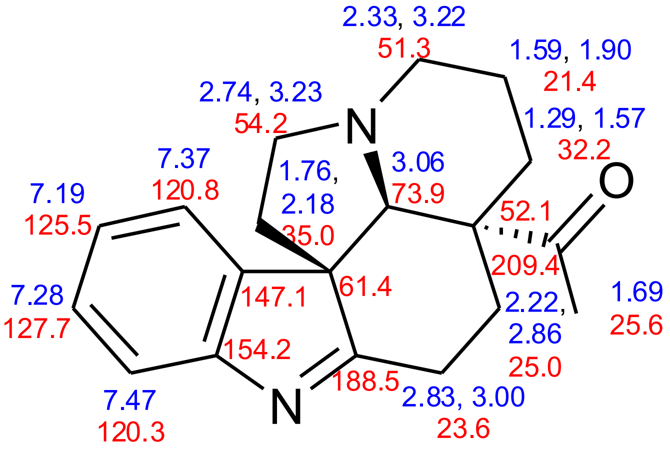
2.33, 3.22
51.3
1.59, 1.90
21.4
2.74, 3.23
54.2
N
1.29, 1.57
32.2
O
7.37
120.8
1.76,
2.18
35.0
3.06
73.9
7.19
125.5
52.1
209.4
147.1
61.4
7.28
127.7
2.22,
2.86
25.0
1.69
25.6
154.2
188.5
2.83, 3.00
23.6
7.47
120.3
N

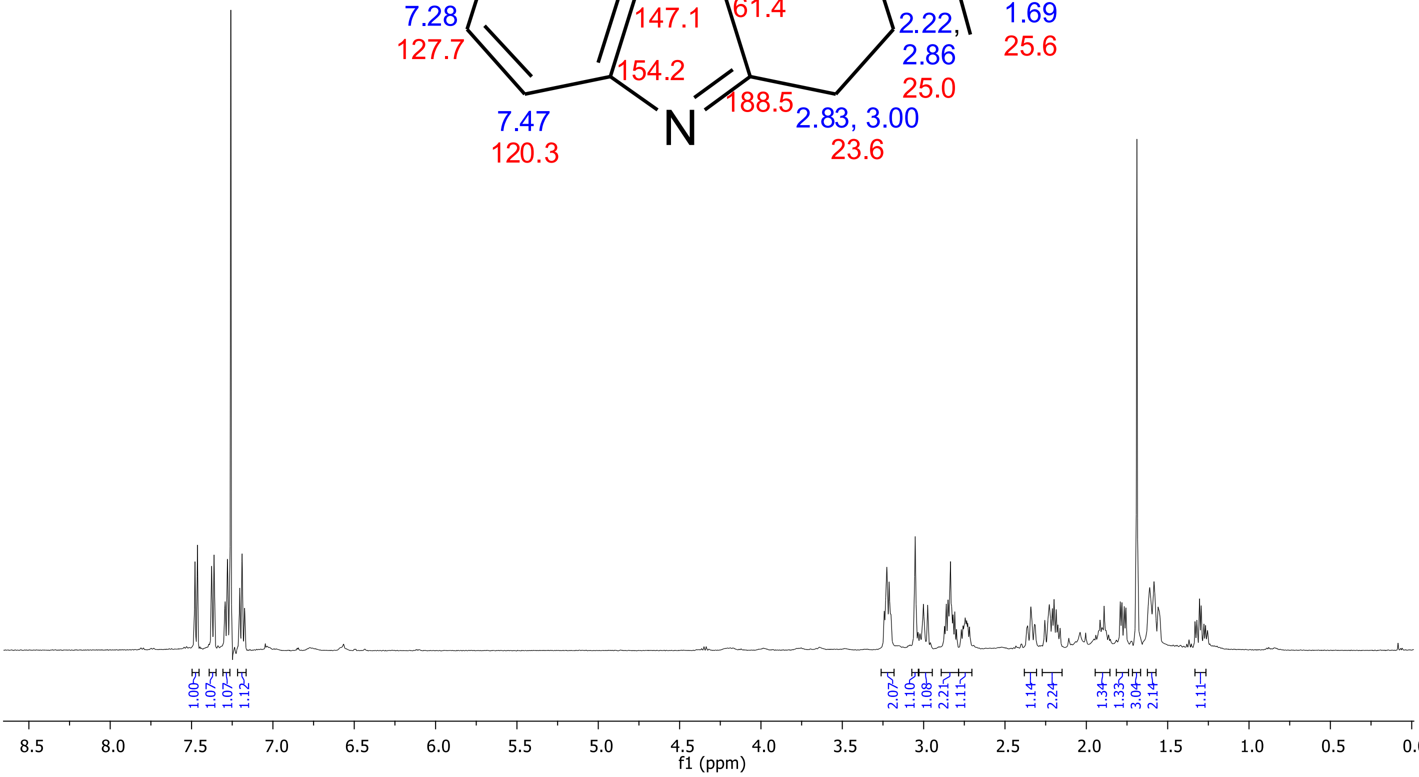
1.00
1.07
1.07
1.12
2.07
1.10
1.08
2.21
1.11
1.14
2.24
1.34
1.33
3.04
2.14
1.11
8.5 8.0 7.5 7.0 6.5 6.0 5.5 5.0 4.5 4.0 3.5 3.0 2.5 2.0 1.5 1.0 0.5 0.0
f1 (ppm)

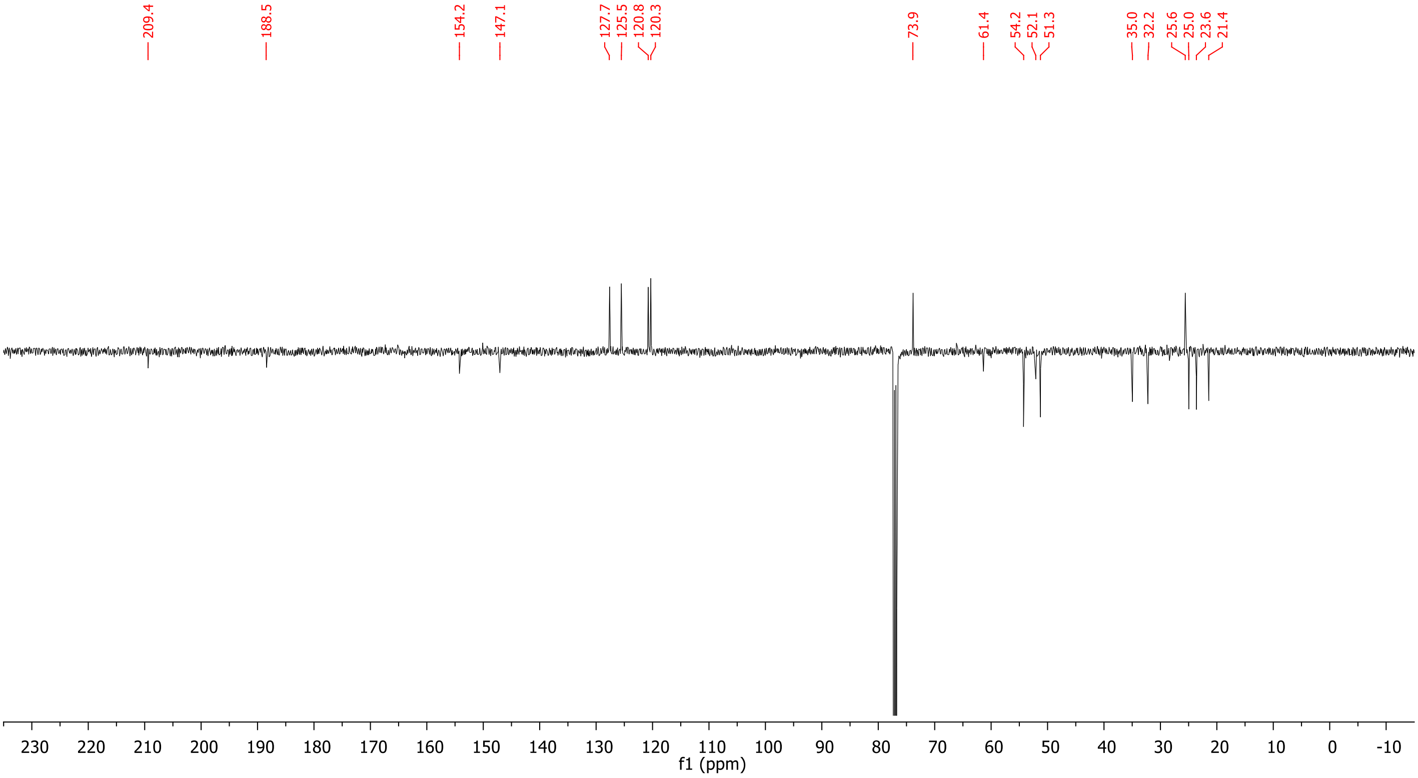
209.4
188.5
154.2
147.1
127.7
125.5
120.8
120.3
73.9
61.4
54.2
52.1
51.3
35.0
32.2
25.6
25.0
23.6
21.4
230 220 210 200 190 180 170 160 150 140 130 120 110 100 90 80 70 60 50 40 30 20 10 0 -10
f1 (ppm)

Synthesis of (-)-aspidofractinine (6)

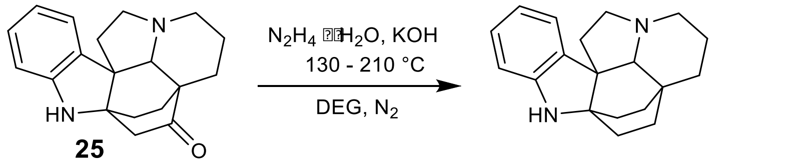

(-)-aspidofractinine (**6**)

Oxo-aspidofractinine **25** (48.0 mg, 0.163 mmol, 1.00 eq.) was dissolved in diethylene glycol (4.0 mL). Hydrazine hydrate (450 µl, 35 m/m%, 30 eq.) and KOH (184 mg, 3.28 mmol, 20.0 equiv.) was added. The flask was flushed with nitrogen and submerged in an oil bath preheated to 130 °C. The mixture was stirred for 1.5 h, when TLC analysis showed no remaining starting material. Then the temperature was raised to 210 °C, resulting in the distillation of volatile compounds, and the mixture was stirred for 16 h. The solution was then treated with distilled water (10 mL), extracted with DCM (3 x 15 mL), dried over Na_2SO_4, filtered and evaporated under reduced pressure. The crude oil was purified by flash column chromatography on flash silica gel (eluted with hexanes/ethyl acetate/Et_3N 2:1:0 then 12:4:1) to afford (-)-aspidofractinine as a transparent oil (40.7 mg, 89% yield).

All analytical data were in accordance with data reported in the literature.[5]

R_f = 0.75 (hexanes/ethyl acetate/ Et_3N 8:8:1) [CAM].

$[\alpha]_D^{23}$ = -18.8 (c = 0.8; $CHCl_3$).

HRMS (ESI): calcd. for $C_{19}H_{25}N_2$ $[M+H]^+$ 281.2018, found 281.2016.

^{1}H NMR ($CDCl_3$, 500 MHz, δ, ppm): 7.34 (1H, dd, *J* = 7.4, 1.2 Hz); 7.00 (1H, td, *J* = 7.4, 1.2 Hz); 6.78 (1H, td, *J* = 7.4, 1.2 Hz); 6.63 (1H, dd, *J* = 7.4, 1.2 Hz); 3.30 (1H, br.s); 3.24 (1H, q, *J* = 8.4 Hz); 3.15 – 3.05 (3H, m); 3.03 (1H, td, *J* = 13.0, 3.1 Hz); 2.72 (1H, ddd, *J* = 13.8, 8.4, 3.6 Hz); 2.30 – 2.05 (2H, m); 1.90 – 1.60 (4H, m); 1.52 (1H, m); 1.42 (1H, m); 1.35 – 1.15 (5H, m).

^{13}C NMR ($CDCl_3$, 125 MHz, δ, ppm): 149.9; 139.8; 126.7; 122.2; 119.8; 110.8; 68.9; 64.4; 57.0; 50.7; 47.8; 35.8; 35.0; 34.6; 31.3 (2C); 29.2; 26.4; 17.0.

[5] D. Gagnon, C. Spino, *J. Org. Chem.* **2009**, *74*, 6035–6041.

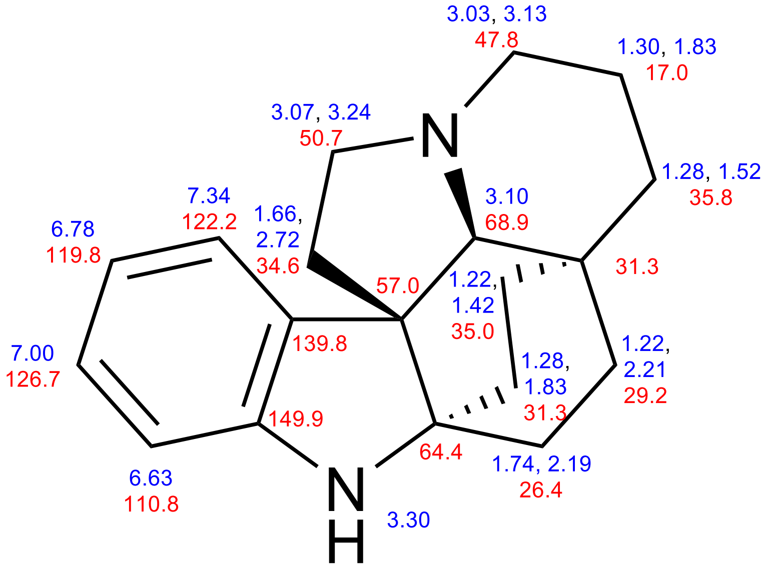
3.03, 3.13
47.8
1.30, 1.83
17.0
3.07, 3.24
50.7
N
1.28, 1.52
35.8
3.10
68.9
7.34
122.2
1.66,
2.72
34.6
6.78
119.8
57.0
1.22,
1.42
35.0
31.3
7.00
126.7
139.8
1.28,
1.83
31.3
1.22,
2.21
29.2
149.9
64.4
1.74, 2.19
26.4
6.63
110.8
N
H
3.30

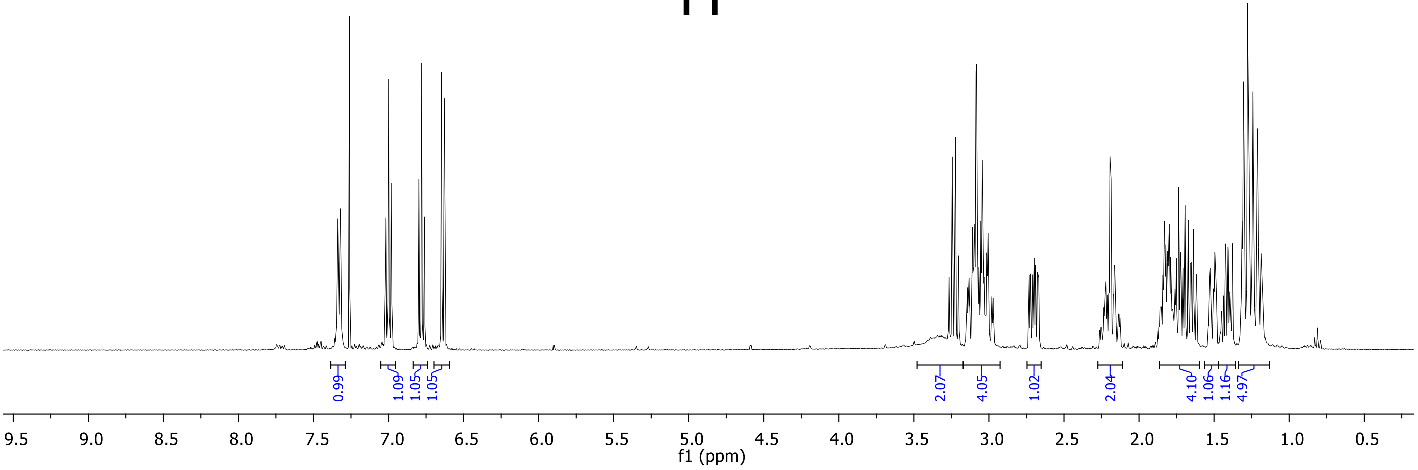
0.99
1.09
1.05
1.05
2.07
4.05
1.02
2.04
4.10
1.06
1.16
4.97
9.5 9.0 8.5 8.0 7.5 7.0 6.5 6.0 5.5 5.0 4.5 4.0 3.5 3.0 2.5 2.0 1.5 1.0 0.5
f1 (ppm)

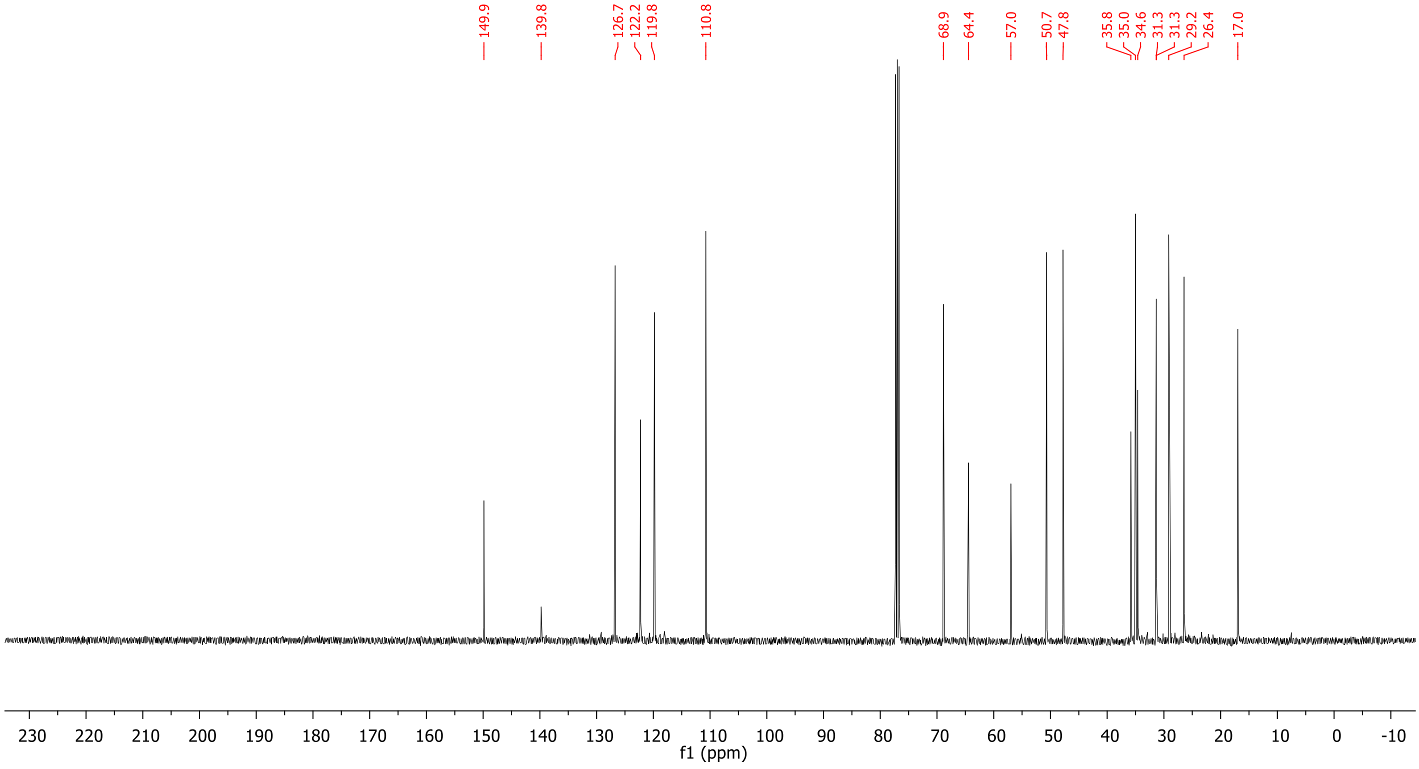
149.9
139.8
126.7
122.2
119.8
110.8
68.9
64.4
57.0
50.7
47.8
35.8
35.0
34.6
31.3
31.3
29.2
26.4
17.0
230 220 210 200 190 180 170 160 150 140 130 120 110 100 90 80 70 60 50 40 30 20 10 0 -10
f1 (ppm)

Optimization of the organocatalytic cascade

Conditions (catalyst, solvent, concentration) were based on our previous optimization[6], however, the optimal catalyst load was further investigated:

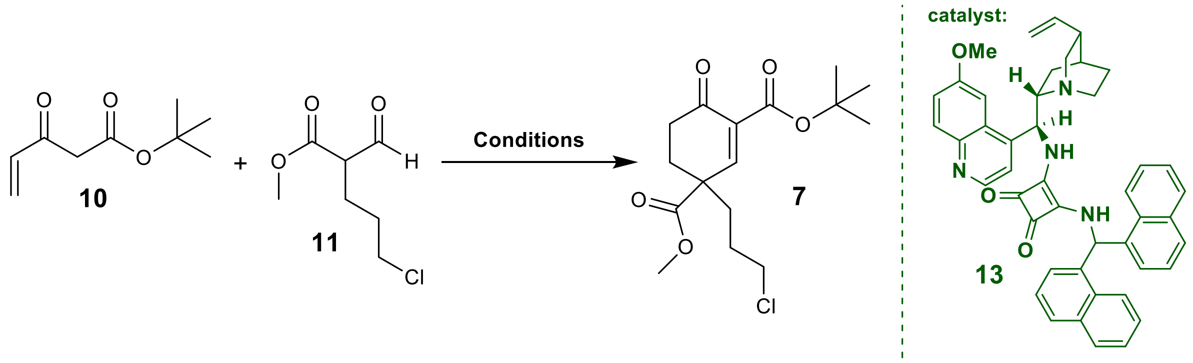

Optimization of the „Large scale" organocatalytic cascade
(Common conditions: catalyst (13), dioxane (1,0 M), room temperature, 6 days)

catalyst load (mol%)	ee (%)	isolated yield of **7** (%)
2	91	71
3	91	77
4	90	76
5	90	77

Results: 3 mol% of catalyst could be used without erosion of the isolated yield or ee.

[6] B. Berkes, K. Ozsváth, L. Molnár, T. Gáti, T. Holczbauer, Gy. Kardos, T. Soós, *Chem. Eur. J.* **2016**, *22*, 18101–18106.

Optimization of the anionic cascade

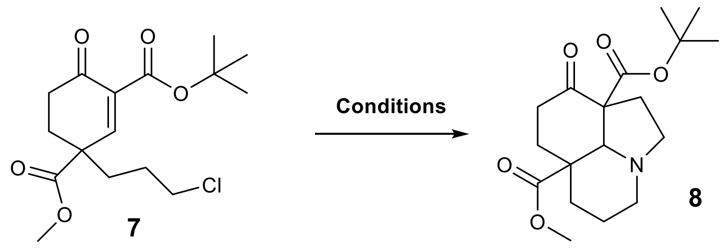

Optimization of the „small scale" anionic cascade
(Common conditions: DCM, room teperature)

aziridine (eq.)	KI (eq.)	2-chloroethylamine hydrochloride (eq.)	DIPEA (eq.)	reaction time (h)	conversion (%)	isolated yield of **8** (%)
10.0	1.1	-	-	2	100	72
5.0	1.1	-	-	4	100	74
5.0	0.1	-	-	48	100	73*
2.5	1.1	-	-	6	100	78
2.05	1.1	-	-	12-16	100	80

*The product mixture contained a side-product (~10%), which was identified by GC-MS as the intermediate **15**.
Results: 1) Yields were not significantly different; 2) the amount of aziridine could be decreased; 3) amount of KI is important to decrease reaction times

Optimization of the „Large scale" anionic cascade
(Common conditions: DCM, room teperature)

aziridine (eq.)	KI (eq.)	2-chloroethylamine hydrochloride (eq.)	DIPEA (eq.)	reaction time (h)	conversion (%)	isolated yield of **8** (%)
-	1.1	1.1	3.1	24	16	14
-	1.1	2.5	5.0	48	100	76
-	1.1	2.5	7.5	16	100	72

Results: 1) The amount of DIPEA is important to increase conversion and decrease reaction time (Rationale: *in situ* generation of aziridine from 2.5 eq. 2-chloroethylamine hydrochloride requires 5 eq. base, substitiution requires further 1 eq. base; overall at least 6 eq. is required to maintain basic conditions)

Isolation and NMR studies on the formation of plausible intermediate 15

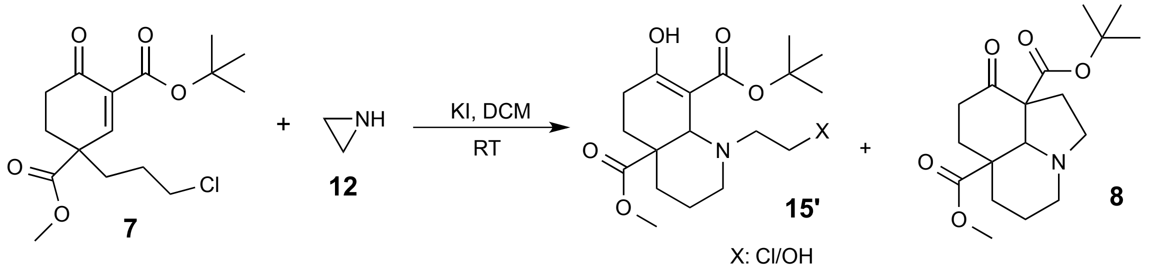

While carrying out and work-up the reaction to produce tricycle **8** among the previously reported "small scale" conditions (for details see S9) we realized a weak (yield < 1%), almost copular spot (**R**$_f$ = 0.50 (hexanes/ethyl acetate 3:1) [$KMnO_4$]) besides the desired product **8** on TLC. Although baseline resolution of the these products was not possible by flash column chromatography (eluted with hexanes/ethyl acetate 5:1), we were able to study a small fraction enriched for intermediate **15'** by NMR (1D and 2D spectras). Further GC/MS study of **15'** enriched mixture gave the same fragmentation pattern for **15'** as for **8**, thus the identity of the X substituent could not be assigned. It could be either Cl or even OH groups (a possible hydrolysis of Cl or I derivatives during work up).

Structure assignment of intermediate 15':

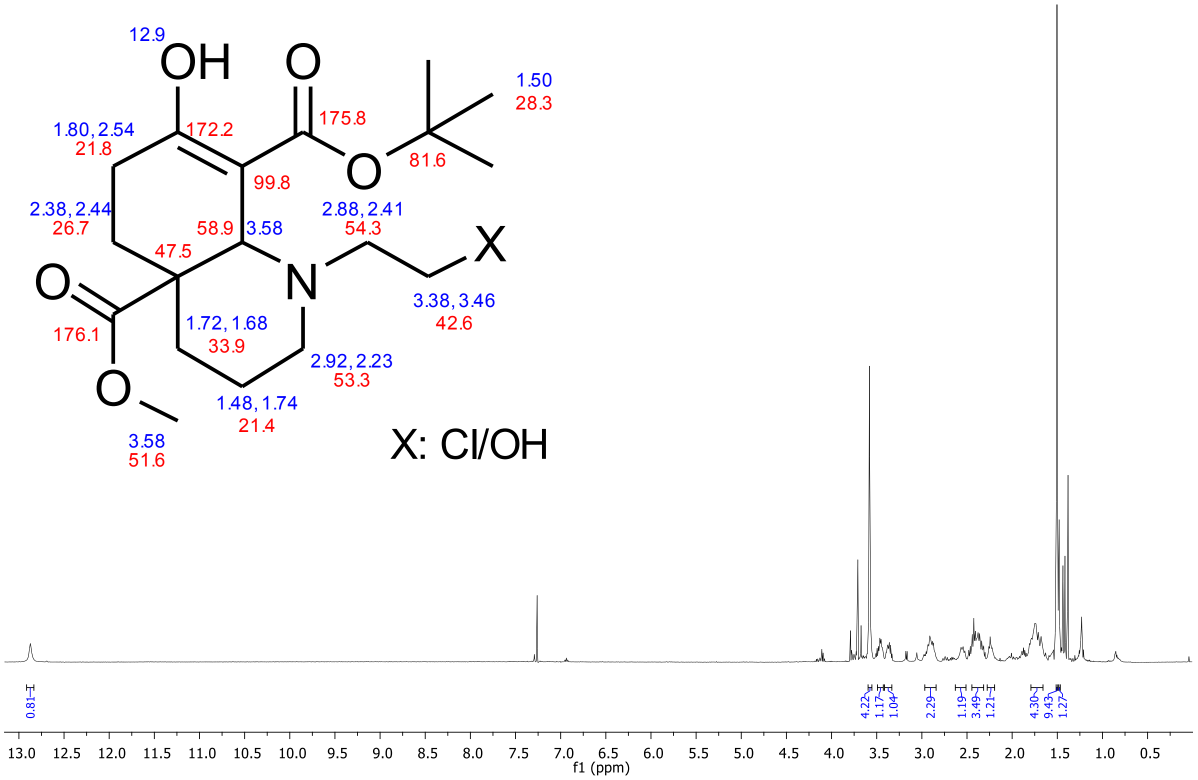

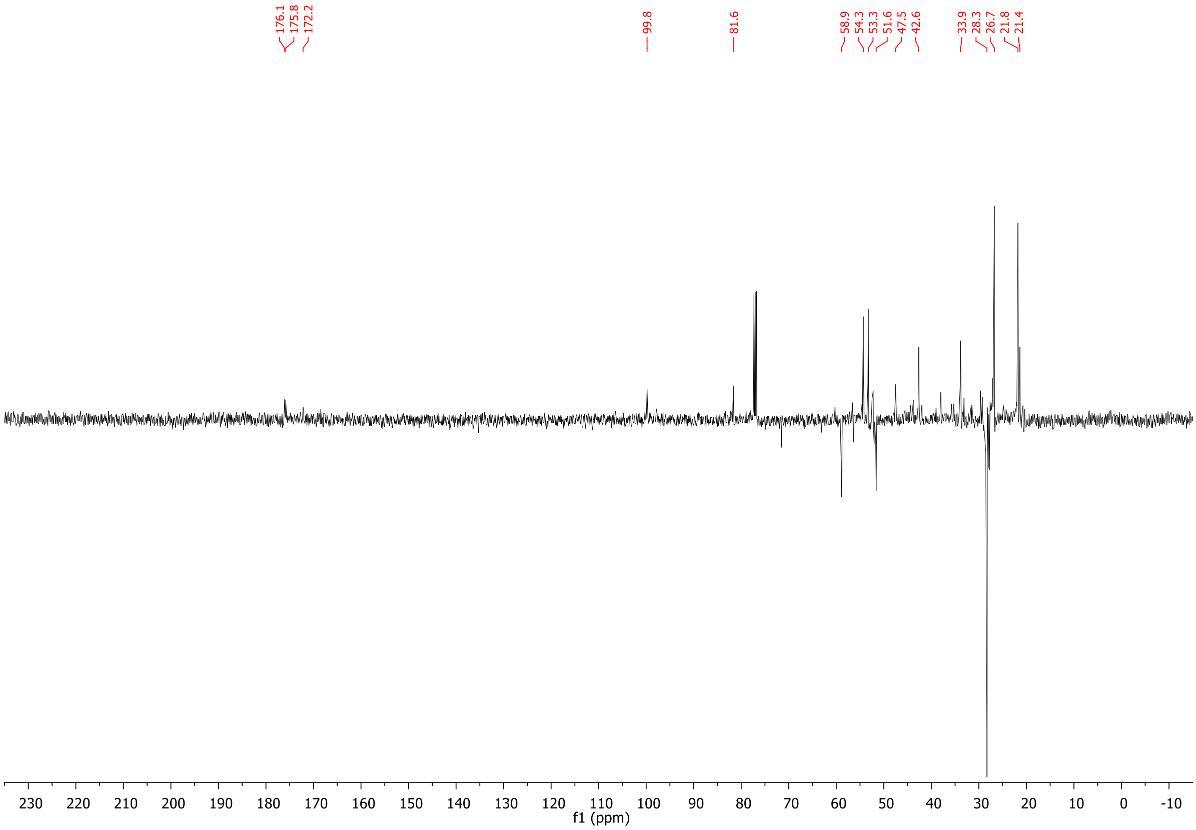
176.1
175.8
172.2
99.8
81.6
58.9
54.3
53.3
51.6
47.5
42.6
33.9
28.3
26.7
21.8
21.4
230 220 210 200 190 180 170 160 150 140 130 120 110 100 90 80 70 60 50 40 30 20 10 0 -10
f1 (ppm)

Synthesis of diketone 23 via ketone 18 - experimental proof of steric shielding

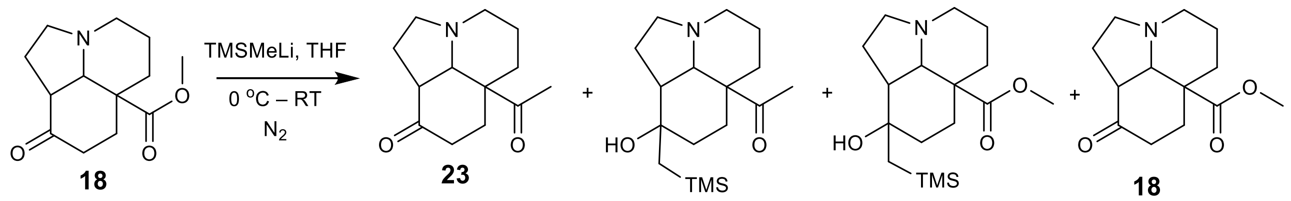

18 → 23 (37%) + 30% + 19% + 18 (14%)

The same procedure utilizing $TMSCH_2Li$, which was used to transform ketone **8** into diketone **23** in two steps (for details see S24) was tested to produce diketone **23** via ketone **18**. GC-MS and NMR analysis of the crude inseparable mixture indicated the formation of products in the ratio depicted above.

Optimization of the last step towards (–)-minovincine

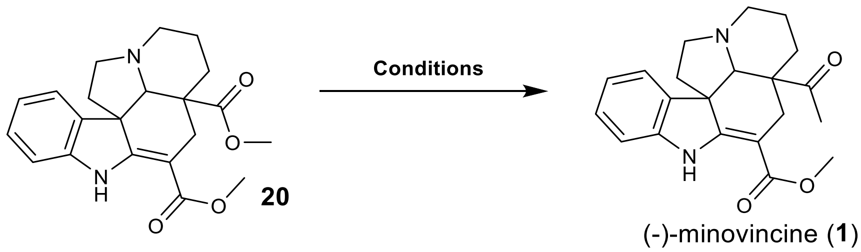

Optimization of the last step towards (–)-minovincine (Common conditions: THF (0.1 M), N_2)

additive (eq.)	reagent (eq.)	temperature (°C)	reaction time (h)	comment	yield of **1*** (%)	unreacted s.m. **20** (%)
-	LDA (1.0)	-78–25	2.5	probing effect of deprotonation	0	45
-	MeLi·LiBr (2.1)	-50–25	3	-	0	60
NaO^{t}Bu (4.1)	$TMSCH_2Li$ (4.1)	-78–25	1+3	warmed to r.t. after 1 h	0	45
-	$TMSCH_2MgCl$ (4.1)	-78–25	1+3	warmed to r.t. after 1 h	0	45
-	$TMSCH_2Li$ (4.1)	25	16	-	traces	45
	$TMSCH_2Li$ (5.5)	25	3	-	30	10
	$TMSCH_2Li$ (7.1)	25	3	-	35	10
	$TMSCH_2Li$ (7.1)	0	3	-	0	55
$BF_3.OEt_2$ (2.0)	$TMSCH_2Li$ (6.1)	-78–25	0.5+3	warmed to r.t. after 0.5 h	25	25
TMEDA (7.1)	$TMSCH_2Li$ (7.1)	25	3		20	0
DABAL-Me_3 (0.5)	$TMSCH_2Li$ (6.1)	-78–25	1+3	1 h adduct formation	40	5
Al^iBu_3 (1.0)	$TMSCH_2Li$ (6.1)	-78–25	1+3	1 h adduct formation	55	5
Al^iBu_3 (1.0)	$TMSCH_2Li$ (3.1)	-78–25	1+3	1 h adduct formation	5>	70
Al^iBu_3 (1.0)	$TMSCH_2Li$ (6.1)	-78–25	1+3	1 h adduct formation, slow addition of reagent	70 (68)**	5

*NMR yield based on internal standard; **isolated yield; Results: 1) simple deprotonation induces decomposition; 2) at least 4 eq. reagent should be used probably due to coordination to Lewis-basic atoms; 3) $TMSCH_2Li$ is reactive only at r.t.; 4) the sterically more hindered Al^iBu_3 is superior to DABAL-Me_3; 5) slow addition of the reagent increases the yield

NMR studies on the formation of adduct 22

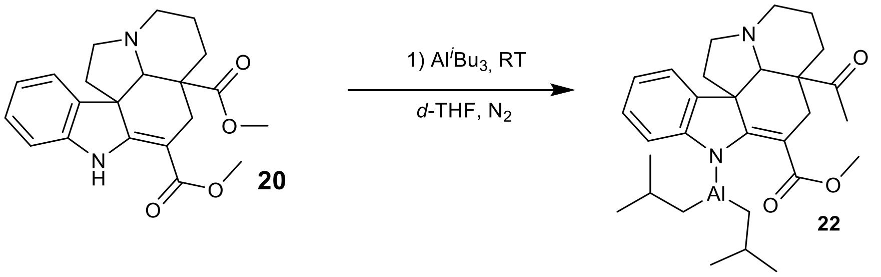

In order to prove the formation of adduct **22** among the previously reported conditions (for details see S22) an NMR experiment was carried out as follows. To a solution of the diester **20** (20 mg, 0.053 mmol, 1.0 eq.) in anhydrous *d*-THF (0.5 mL) was added Al^{i}Bu$_3$ in toluene (0.1 mL, 1.0 M, 2.0 eq.) at RT under nitrogen atmosphere. A change to bright yellow colour was immediately experienced and shift in ^{13}C-NMR spectra was investigated. The shift pattern indicated that the aluminum has complexed to N-1 and not N-9 or any other Lewis-basic atoms (e.g. oxygen in esters).

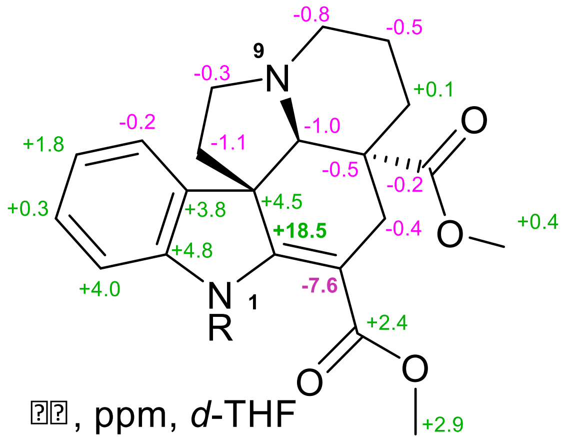

[illegible], ppm, *d*-THF

Chiral HPLC data – building block 7

Enantioselectivity was determined by HPLC analysis with a Chiralpak IC 250x4.6 mm, 5 μm column, 10% isopropyl alcohol in hexanes, 1.0 mL/min, T = 20 °C, λ = 254 nm, t_r(minor) = 19.8 min, t_r(major) = 24.2 min.

Small scale (reaction aa, 90% ee)

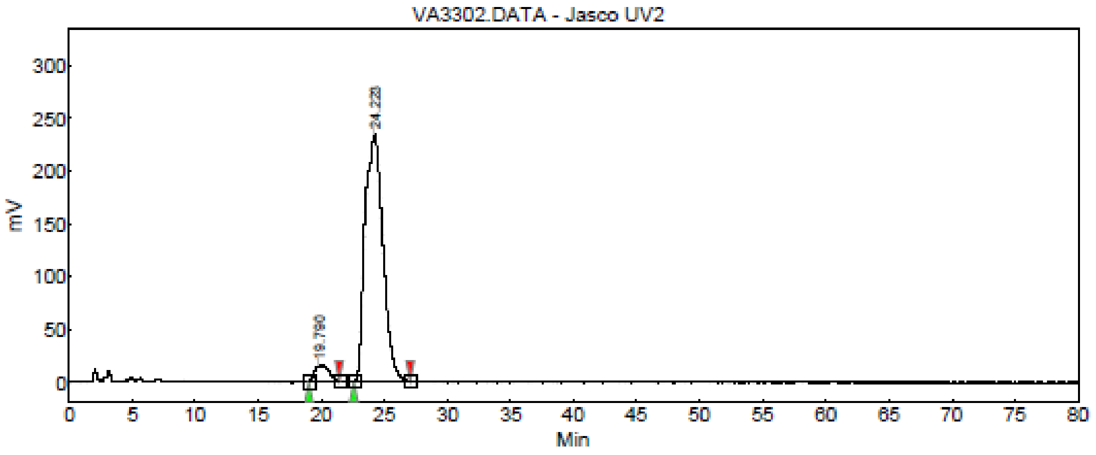

Index	Name	Time [Min]	Quantity [% Area]	Height [mV]	Area [mV.Min]	Area % [%]
1	UNKNOWN	19.790	4.86	15.6	20.0	4.862
2	UNKNOWN	24.223	95.14	234.3	392.3	95.138
Total			100.00	249.9	412.3	100.000

Large scale (reaction ab, 91% ee)

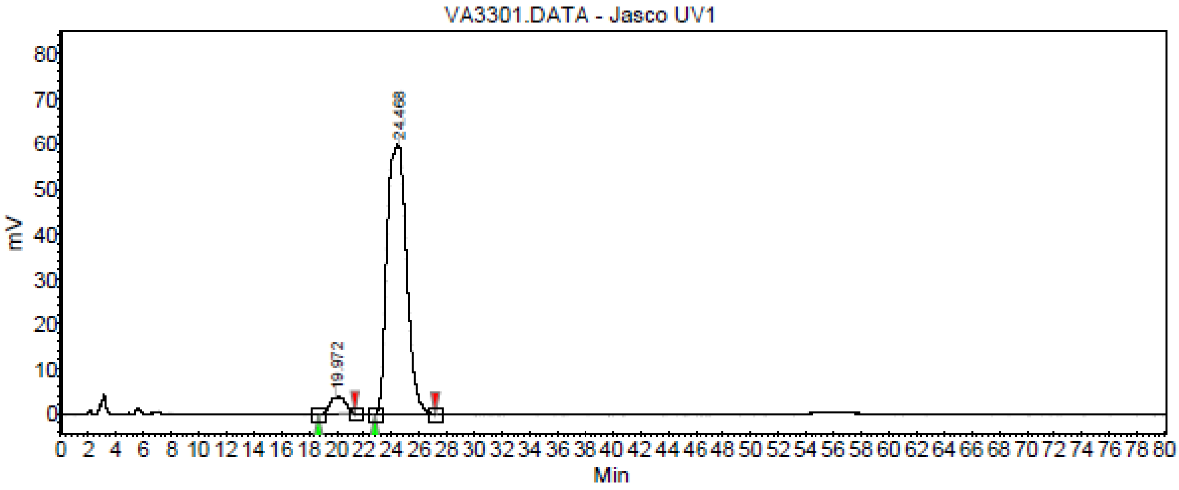

Index	Name	Time [Min]	Quantity [% Area]	Height [mV]	Area [mV.Min]	Area % [%]
1	UNKNOWN	19.972	4.60	3.8	4.8	4.603
2	UNKNOWN	24.468	95.40	60.0	99.7	95.397
Total			100.00	63.8	104.5	100.000

Racemic

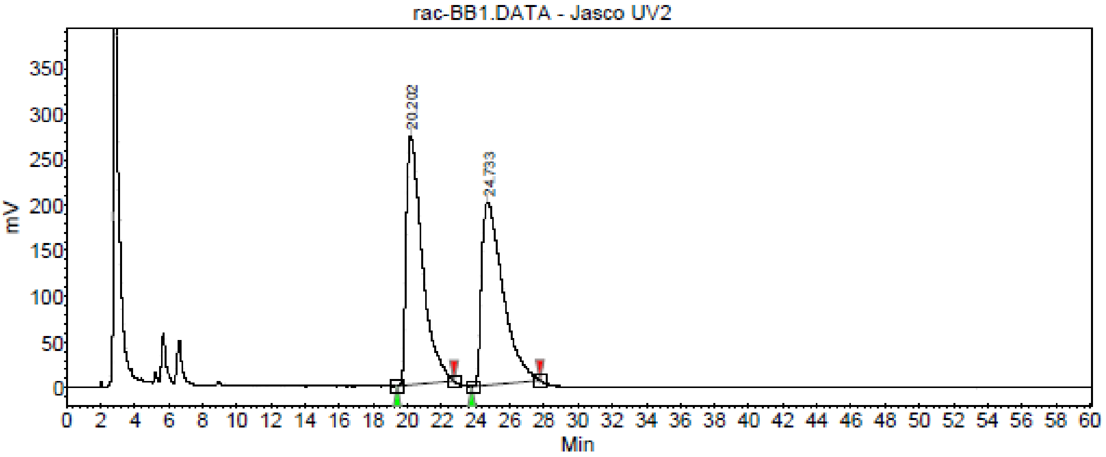

Index	Name	Time [Min]	Quantity [% Area]	Height [mV]	Area [mV.Min]	Area % [%]
1	UNKNOWN	20.202	50.77	274.7	299.9	50.767
2	UNKNOWN	24.733	49.23	201.0	290.8	49.233
Total			100.00	475.7	590.7	100.000

X-ray data – tricycle 8

Crystal data: C18 H27 N O5, *Fwt.*: 337.40, colourless, chunk, size: 0.500 x 0.400 x 0.300 mm, monoclinic, space group *P* 21, $a = 9.9745(5)$Å, $b = 13.9875(7)$Å, $c = 12.7170(6)$Å, $\alpha = 90°$, $\beta = 92.272(7)°$, $\gamma = 90°$, $V = 1772.86(15)$Å^3, $T = 103(2)$K, $Z = 4$, $F(000) = 728$, $D_x = 1.264$ Mg/m^3, μ 0.751mm^{-1}.
A crystal of **8** was mounted on a fiber. Cell parameters were determined by least-squares using 20358 ($3.16 \le \theta \le 68.195°$) reflections.
Intensity data were collected on a) Rigaku R-AXIS-RAPID II diffractometer (monochromator; Cu-$K\alpha$ radiation, $\lambda = 1.54187$Å) at 103(2) K in the range $3.478 \le \theta \le 68.236$.[7] A total of 22641 reflections were collected of which 6228 were unique [R(int) = 0.0295, $R(\sigma) = 0.0263$]; intensities of 6018 reflections were greater than $2\sigma(I)$. Completeness to $\theta = 0.992$.
A numerical absorption correction was applied to the data (the minimum and maximum transmission factors were 0.929574 and 0.952941).
The structure was solved by direct methods[8] (and subsequent difference syntheses).
Anisotropic full-matrix least-squares refinement[9] on F^2 for all non-hydrogen atoms yielded $R_1 = 0.0296$ and $wR^2 = 0.0711$ for 1332 [$I > 2\sigma(I)$] and $R_1 = 0.0309$ and $wR^2 = 0.0718$ for all (6228) intensity data, (number of parameters = 441, goodness-of-fit = 1.034, the maximum and mean shift/esd is 0.000 and 0.000). The absolute structure parameters are (Flack x:) 0.10(4); (Hooft y:) 0.08(4); (Parsons z:) 0.09(3). (Friedel coverage: 0.857, Friedel fraction

[7] CrystalClear SM 1.4.0 (Rigaku/MSC Inc., **2008**).

[8] NUMABS: Higashi, T. (**1998**), rev. **2002**. (Rigaku/MSC Inc.)

[9] G.M. Sheldrick, Acta Cryst. **2008**, A64, 112-122.

max.: 0.929, Friedel fraction full: 0.933).
The maximum and minimum residual electron density in the final difference map was 0.20 and -0.14e.Å^{-3}.

The weighting scheme applied was $w = 1/[\sigma^2(F_o^2)+(0.03450.3438P)^2+0.3438P]$ where $P = (F_o^2+2F_c^2)/3$.
Hydrogen atomic positions were calculated from assumed geometries. Hydrogen atoms were included in structure factor calculations but they were not refined. The isotropic displacement parameters of the hydrogen atoms were approximated from the *U*(eq) value of the atom they were bonded to. The configuration of C3A, C6A C9A atoms in the two independent molecule are in order: *S*, *R*, *R*. ORTEP style molecular structure diagram can be found in Figure S1 while crystallographic data are in Table S1.
Crystallographic data (including structure factors) for the crystal structure of **8** has been deposited with the Cambridge Crystallographic Data Centre as supplementary publication number CCDC 1988772.

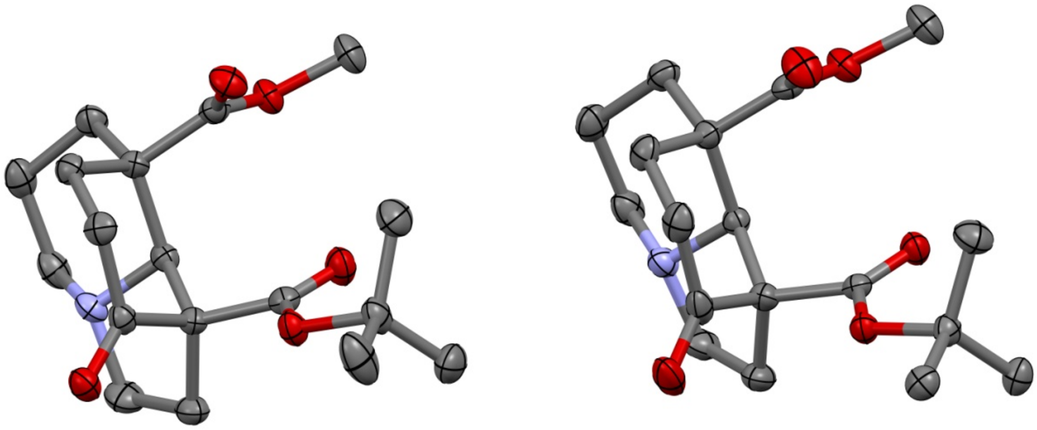

Fig S1. ORTEP style representation of the two crystallographically independent molecules in the asymmetric unit without hydrogen atoms. The molecules have the same configuration (C3A, C6A, C9A: *S*, *R*, *R*). Displacement ellipsoids are drawn at the 50% probability level.

Table S1: Summary of crystallographic data, data collections, structure determination and refinement for **8**

Empirical formula	$C_{18}H_{27}N_1O_5$
Formula weight	337.40
Temperature	103(2)
Radiation	Cu-Kα,
wavelength λ (Å)	1.54187
Crystal system	monoclinic
Space group	$P2_1$
Unit cell dimensions:	
a (Å)	9.9745(5)
b (Å)	13.9875(7)
c (Å)	12.7170(6)
α (°)	90
β (°)	92.272(7)
γ (°)	90
Volume (Å^3)	1772.9(2)
Z, Z'	4, 2
Density (calculated) (Mg/m^3)	1.264
Absorption coefficient, μ(mm^{-1})	0.751
F(000)	728
Crystal colour	colourless
Crystal description	chunk
Crystal size (mm)	0.50 x 0.40 x 0.30

Absorption correction	numerical
Max. and min. transmission	0.929574 and 0.952941
θ–range for data collection (°)	$3.478 \leq \theta \leq 68.236$
Index ranges	$-12 \leq h \leq 11$; $-16 \leq k \leq 16$; $-15 \leq l \leq 15$
Reflections collected	22641
Completeness to 2θ	0.993
Absolute structure parameters:	
Flack x	0.10(4)
Hooft y	0.08(3)
Parsons z	0.09(3)
Friedel coverage	0.857
Friedel fraction max.	0.929
Friedel fraction full	0.933
Independent reflections	6228 [*R*(int) =0.0295]
Reflections $I > 2\sigma(I)$	6018
Refinement method	full-matrix least-squares on F^2
Data / restraints / parameters	6228 /1 /441
Goodness-of-fit on F^2	1.034
Final *R* indices [$I > 2\sigma(I)$]	R_1 =0.0296, wR^2 =0.0711
R indices (all data)	R_1 =0.0309, wR^2 =0.0718
Max. and mean shift/esd	0.000;0.000
Largest diff. peak and hole (e.Å^{-3})	0.20 and -0.14
